# Improving Activity and Stability of *Candida boidinii* Formate Dehydrogenase Through Rational Active Site Engineering

**DOI:** 10.1002/cbic.70497

**Published:** 2026-08-14

**Authors:** Marisa Bickmann, Andrea Rodil, Jan Deska

**Affiliations:** ^1^ Department of Chemistry University of Helsinki Helsinki Finland; ^2^ Institute for Bioorganic Chemistry Heinrich‐Heine‐University Düsseldorf Jülich Germany

**Keywords:** biocatalysis, cofactor recycling, formate dehydrogenase, NADH, protein engineering, rational design

## Abstract

Formate dehydrogenases represent a highly attractive tool for nicotinamide cofactor regeneration to drive biocatalytic reductions and oxygenations by NADH‐dependent oxidoreductases with formate as a cheap, atom‐economic terminal reductant. However, despite the clear benefits over other recycling systems, native FDHs remain rather unpopular, mainly due to their intrinsic, low specific activity. While the active site architecture is generally highly conserved across many FDHs from very diverse origins, smaller patterns of dissonance in the active site's vicinity can serve as hotspots for the rational design of more effective variants. In this study, we have created a series of *Candida boidinii* FDH variants incorporating nonconsensus amino acids found in *Saccharomyces cerevisiae* FDH. A double variant (C23S/F285D) was identified that exhibits a significantly higher specific activity, an overall more desirable pH and temperature profile, and an improved stability profile.

## Graphical abstract

In search of improved biocatalysts for the NADH cofactor recycling in synthetic reductive biotransformations, *C. boidinii* formate dehydrogenase is the subject of a rational protein engineering approach. The study delivers an enzyme variant with improved specific activities and a more desirable pH and temperature profile, which shows promising performance in both ene‐ and keto‐reductions.

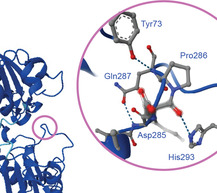

## 1 Introduction

Nicotinamide adenine dinucleotide (NAD^+^), and its phosphorylated counterpart (NADP^+^), are essential coenzymes in a myriad of biochemical processes. From its physiological relevance [1, 4] to its role in laboratory‐developed synthetic protocols [5, 8], it is fairly obvious that the NAD(P)^+^/NAD(P)H redox couples are not just ubiquitous in the context of enzyme catalysis but likely the most central cofactors across biological chemistry. However, the most efficient and economical way of employing these cofactors is by optimizing a recycling system. In fact, the regeneration of NADH has its own niche in research [9, 12], along with the development of cofactor analogs [13, 17].

Popular protocols for the effective in vitro recycling of nicotinamide coenzymes include the use of chemical reductants, electrochemical cofactor manipulation, as well as photocatalytic and enzymatic approaches [9]. Although these methods might seem far away from each other, in truth, most of them are not standalone and rely on secondary complementary systems to work. For example, the catalytic reduction of NAD(P)^+^ with transition metal catalysts (Rh, Ru, Ir, Fe) is often combined with the enzymatic reduction of CO_2_ [9]; whilst the use of flavin mononucleotide as a recycling agent [18] has recently been enhanced by coupling with a flavin oxidoreductase [19]. Photochemical and electrochemical cases have been described by mimicking the natural pathway of electron transfer via ferredoxin and ferredoxin reductase [20, 21], using graphitic carbon nitride [22] or by a host–guest style recognition mediated by cucurbit [8]uril on nickel oxide [23], amongst many other examples [24, 27]. In spite of the growing research on these methods, the enzymatic approach to cofactor recycling can still be considered the greenest, most selective, with a lower environmental impact, and in general, most efficient [26, 28, 29].

In most instances, enzymatic recovery of NAD^+^/NADH occurs through dehydrogenases [28, 30]. Alcohol [31, 32], glucose [33, 34], lactate [35, 36] or formate dehydrogenases [28, 37, 38] are typical choices for this purpose, and they all present advantages and disadvantages [39, 41]. Overall, it can be argued that formate dehydrogenase (FDHs) is the best option, taking into account different factors [42]. In the first place, these enzymes catalyze the oxidation of formate, a cheap and abundant substrate, to produce carbon dioxide via NAD^+^ reduction. The product, CO_2_, is kinetically and thermodynamically stable and volatile, preventing the modification of reaction conditions that could affect the catalytic activity, as well as the formation of unwanted side products [43, 44]. Second, it has been shown that the coproduction of FDH with other enzymes improves the availability of intracellular NADH, resulting in higher enzymatic productivity [45]. Finally, FDHs are usually more atom‐economic, and FDH‐driven recycling does not come with the accumulation of large amounts of organic coproducts [46]. In general terms, formate dehydrogenases are a very heterogeneous group of enzymes [47], but their main form of classification relies on the absence or presence of molybdenum‐ or tungsten‐based metal centers in the active site. Nonmetallo FDHs usually require NAD(P)H as cofactors [48], and this article will focus specifically, and for obvious reasons, on them.

The particular case of NAD^+^‐dependent formate dehydrogenases is curious: while there are plenty of studies highlighting their aptitude for cofactor recycling [37, 49, 51], the reality is that FDH has an intrinsically low activity and catalytic efficiency, as well as limited stability [52, 53]. The only commercial option, from *Candida boidinii* (*Cb*FDH), is often used as a model yeast enzyme [54, 56], but it is still far from providing optimum results, and other FDHs are being explored [57, 59]. With this article, we hope to contribute to the considerable effort made by many to improve the innate characteristics of *Cb*FDH.

## 2 Results and Discussion

### 2.1 Design of New Variants

Although directed evolution and error‐prone Polymerase chain reaction (PCR) can be incredibly powerful tools for the efficient creation of mutant libraries, they can also be time‐consuming, and they require a high‐throughput method for analysis. Instead, rational design can be used with precision to develop specific variants that could give the desired results, saving both time and resources. Moreover, the precise and targeted modulation of preselected amino acids allows for rapid elucidation of hypotheses regarding structure–activity relationships to build the knowledge basis for informed protein engineering campaigns.

**FIGURE 1:**
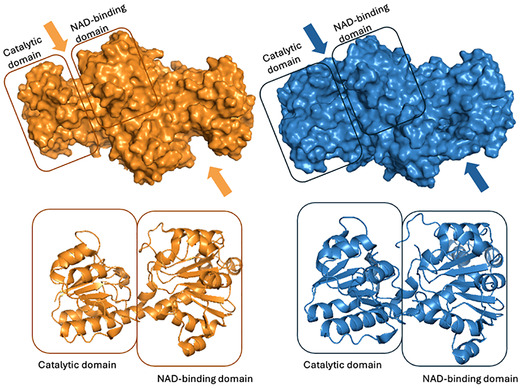
Apo (orange, PDB:5DNA) and holo (blue, PDB:5DN9) structures of *Candida boidinii* FDH [64]. Their active site channel, as marked by the arrows, between the catalytic domain and the NAD^+^‐binding domain of the enzyme. This channel remains open in the absence of binding, but closes to enable catalysis, leaving the catalytic site buried within the structure. This figure was created with PyMol [65].

NAD^+^‐dependent FDHs are characterized for being extremely well‐conserved, resulting in a high degree of homology between species [60, 62]. Much like the FDH in *Pseudomonas* sp. 101 (*Ps*FDH), *Cb*FDH has a long active site channel that ends at the enzymatic substrate‐binding site, and it is placed in the cleft between the catalytic and the NAD^+^‐binding domains [63]. Interestingly, this channel is visibly open in the apo‐enzyme (orange), but closes down once the active site has been occupied (holo‐enzyme, blue), as shown in Figure 1 [64]. This closure has been reported to be an inescapable prerequisite for catalysis to occur, given that otherwise, the surrounding water molecules would impede the hydride transfer necessary for the reaction to go forward [63, 66]. Once the channel is modified post‐binding, the catalytic site is buried within the enzymatic structure.

The similarities between *Ps*FDH and *Cb*FDH are striking, and a great deal of the understanding on *Cb*FDH to this day was gathered by inference from the formate dehydrogenase in *Pseudomonas* sp. 101. Not in vain, as their active sites are highly conserved and seem to behave in an extremely similar manner [54, 62, 64]. In fact, the catalytic and substrate binding site in *Cb*FDH has been shown to include the same residues as *Ps*FDH, with the exception of V93, which in the case of the *Pseudomonas* enzyme is I122 [64, 67]. Another difference between these enzymes is that *Ps*FDH carries a higher number of cysteines. *Cb*FDH contains only two, one directly on the surface (C23) and one buried within (C262) [68]. *Cb*C23 corresponds to a serine in *Ps*FDH. Importantly, the C23S mutation in *C. boidinii* FDH proved to have a generally stabilizing effect, and it has therefore been used widely in the literature as a starting point in FDH engineering campaigns [56, 68, 70]. Consequently, C23S was adopted as a base mutation for our own variants.

**FIGURE 2:**
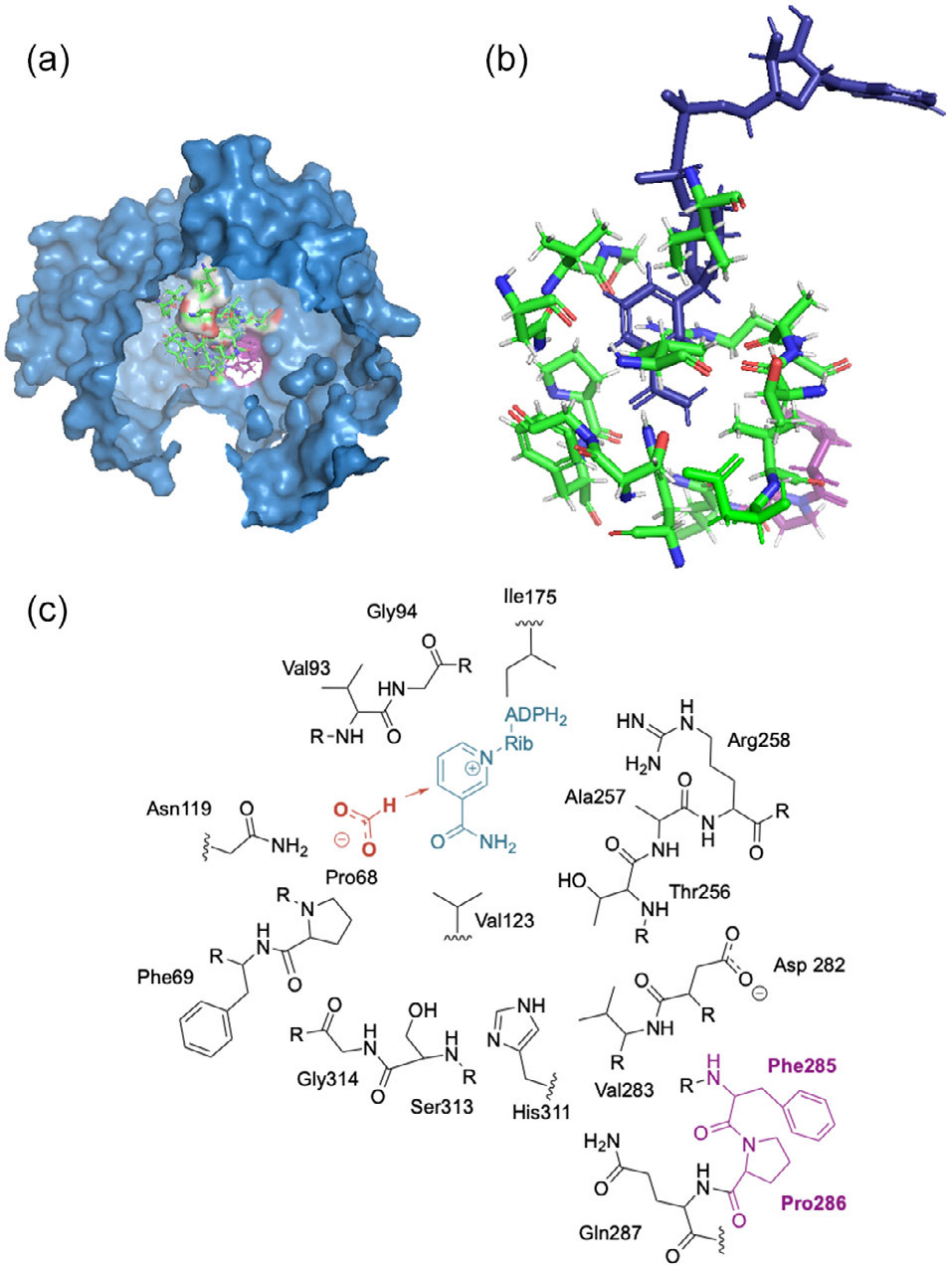
Active site of *Cb*FDH buried within the subunit A of the protein (a) and by itself (b). The entrance from the substrate channel is from the back, as the light purple arrow shows, coming from right above Phe285 and Pro286 (marked purple). (c) Is a representation of the active site from the same perspective as (b).

**FIGURE 3:**
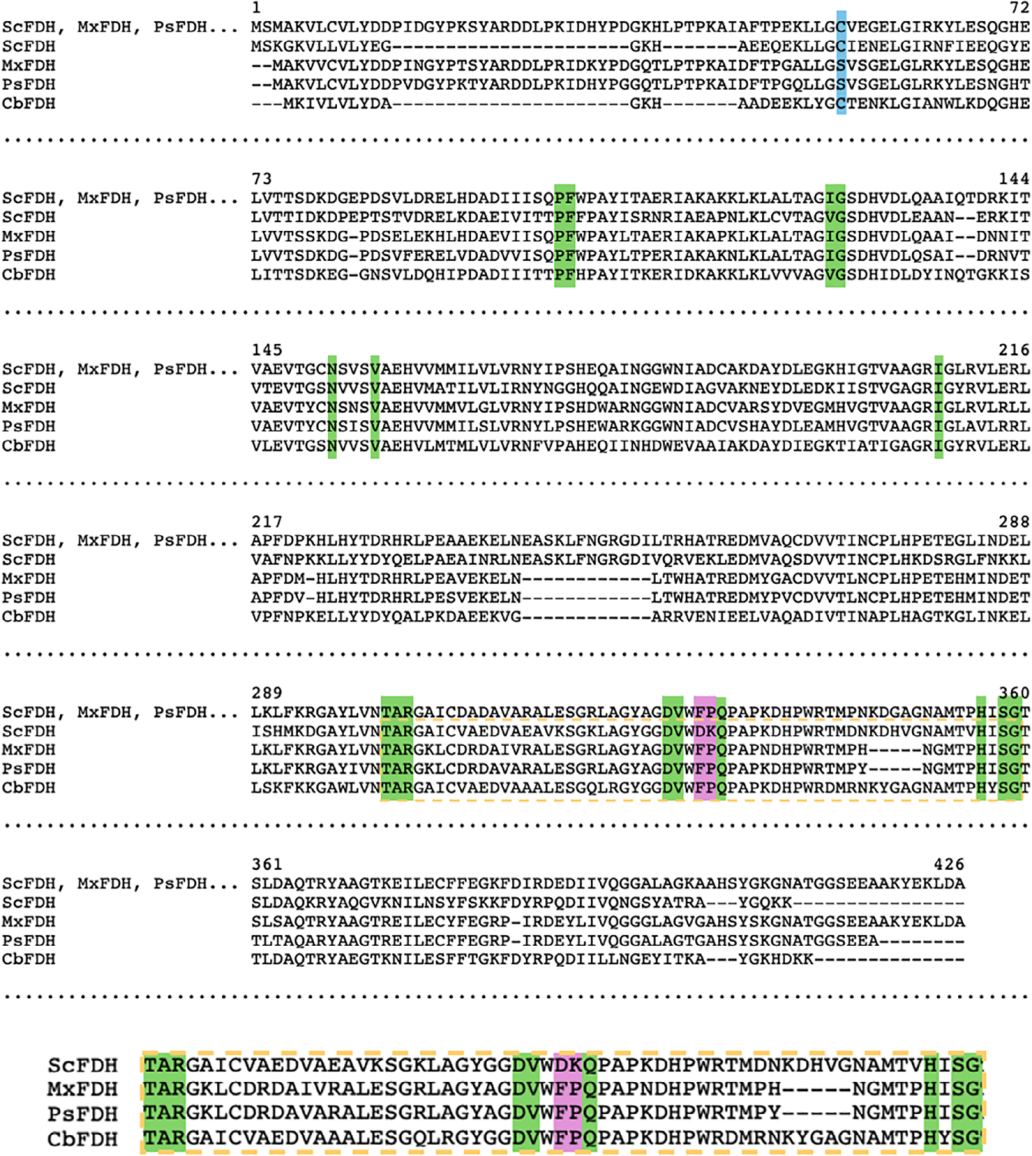
Amino acid sequence alignment of the formate dehydrogenases in *S. cerevisiae* (*Sc*FDH; UniProt Q08911), *Moraxella* sp. (*Mx*FDH; UniProt A0ACD6B9E8), *Pseudomonas* sp. (*Ps*FDH; UniProt P33160) and *C. boidinii* (*Cb*FDH; UniProt A0A0A1EQY0). Highlighted in blue is the base mutation site CbC23, and in purple are the target sites for our rational design, *Cb*F285 and *Cb*P286. The green squares show amino acids directly involved in catalysis. The top row of the alignment corresponds to the consensus sequence between all four species. The dotted square below displays an enlarged image of our mutation site of interest, as well as nearby active site residues.

On the other hand, the consensus approach to rational design suggests that direct substitution of any of these highly conserved residues could easily prove counterproductive given their crucial roles in catalysis; however, nearby locations that do not actively participate in catalysis but have secondary interactions that facilitate the process might be optimal targets for site‐directed mutagenesis. Sequence alignment with other NAD^+^‐dependent FDHs shows that F285 is located in a highly conserved area, just upstream of D282 and V283, and downstream of a glutamine moiety (Q287), all of which are directly involved in catalysis (Figures 2 and 3). F285 also happens to be right next to the channel “lid”. In fact, in an alignment done by Tishkov and Popov (2004), which includes FDHs from bacteria, proteobacteria, yeasts, fungi, higher plants and mammals, it becomes apparent that F285 is a feature in all sequences, except for four: potato favors tyrosine (similar to *C. boidinii*'s phenylalanine) but yeast *S. cerevisiae*, fungi *G. zeae* PH‐1 and plant *A. thaliana* all contain aspartic acid, a considerably different, charged structure [59].

Previous directed evolution studies using error‐prone PCR discovered that variant F285S presented improved enzymatic activity. The author of this study hypothesises that substituting the phenylalanine with a serine creates a “mis‐interaction” of the serine with the channel “lid”, resulting in faster substrate binding and product release [71]. It has also been determined that F285 has an effect upon *C. boidinii*'s FDH thermal and structural stability [72]. These separate pieces of research further support our hypothesis that the phenylalanine in position 285 holds key interactions toward catalytic function, and reassured us in our endeavor. Successive sequential analysis of this relevant area in different species brings to light a second detail: *Saccharomyces cerevisiae* FDH (*Sc*FDH) contains a lysine where *Cb*FDH harbors proline (position 286, Figure 3). Given that recombinant *Sc*FDH has traditionally exhibited higher specific activity than *Cb*FDH, this observation could be important for the development of an improved FDH [59, 68, 73].

Thus, the exploration of sites 285 and 286 seemed like a natural choice to attempt rational design site‐directed mutagenesis following the nonconsensus recipe identified in *Saccharomyces*. In effect, we have explored three novel variants to *Cb*FDH: C23S/F285D, C23S/P286K and C23S/F285D/P286K, to assess the relevance of these sites toward the functionality of *C. boidinii* formate dehydrogenase, as well as their structural role in the catalytic channel, by the potential interaction with the amino acids at its entrance.

Codon‐optimized genes for *Cb*FDH (sequence by Guo et al.) [64] and *Cb*FDH‐C23S/F285D/P286K were purchased from Eurofins Genomics and inserted into the expression vector pET‐28a(+). By contrast, *Cb*FDH‐C23S, *Cb*FDH‐C23S/F285D and *Cb*FDH‐C23S/P286K were created in situ by parallel “SPRINP” PCR‐based site‐directed mutagenesis to *Cb*FDH [74]. The modified genes were subsequently transformed into competent *Escherichia coli* BL21 DE3 cells. Enzyme‐encoding genes were overexpressed with IPTG and purified using standard IMAC methods, with a Ni‐NTA chromatographic column (see Experimental Methods and Supporting Information for a detailed description of these steps).

### 2.2 Activity and Stability Profiles

**FIGURE 4:**
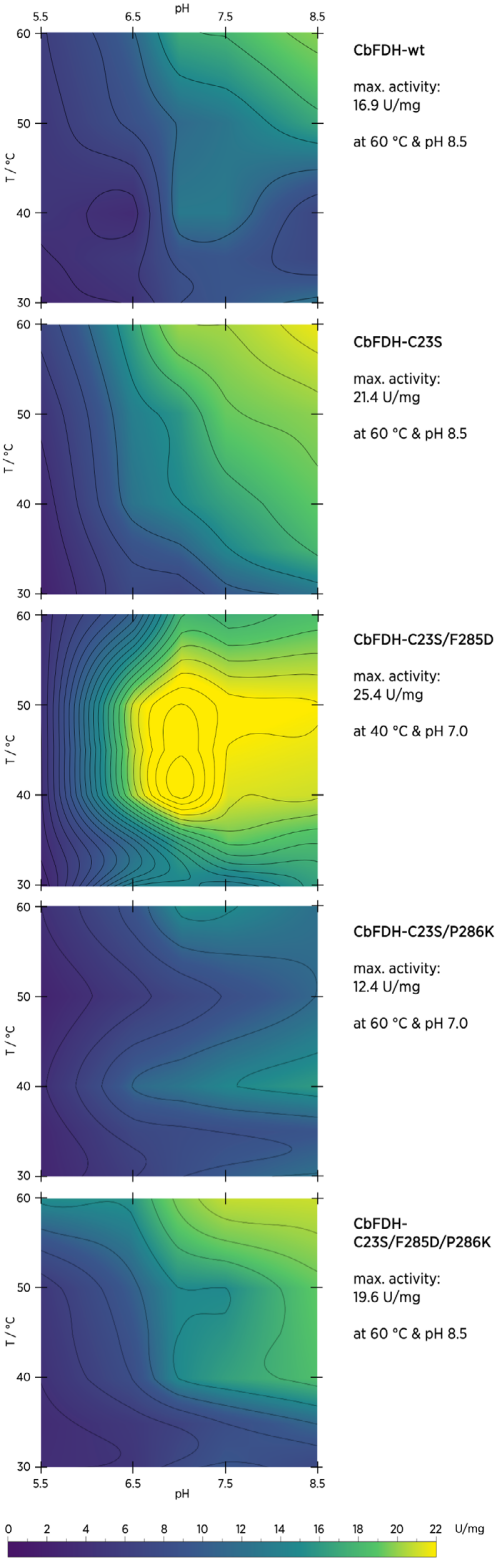
Temperature and pH profiles of wild‐type *Cb*FDH and the four investigated variants.

One of the commonly cited advantages of enzymatic reactions is their ability to carry out complex chemistry in very mild conditions, namely 30–40 °C and neutral pH. The activity response of our enzymes was established with the same set of experiments designed to provide information on the pH and thermal profiles of the new variants compared to recombinant wild type. We tested enzymatic catalysis at pH 5.5, 6.5, 7, 7.5, and 8.5 at 30, 37, 40, 50, and 60 °C, and compiled the results in Figure 4.

According to our data, recombinant wild‐type FDH shows a natural tendency toward more basic pHs, which seems to also be consistent with other reports [53, 75]. This alkaline tendency is further evidenced when looking at the full pH and temperature profile, as it clearly shows that most of the variants analyzed have their optimal results at pH 8.5. To this end, it can be said that variants C23S and C23S/F285D/P286K conserve this characteristic. C23S/F285D, however, has its optimum at pH 7, something that is replicated in C23S/P286K.

The temperature profile equally follows this pattern. Wild‐type, C23S and C23S/F285D/P286K all have their peak at 60 °C, and in this case, so does C23S/P286K. Once again, C23S/F285D stands apart by showing the highest activity at 40 °C. The fact that this double variant thrives at a neutral pH and much lower temperature than its counterparts makes it highly attractive as an NAD^+^ recycling system under the mild conditions that most other enzymes involved in the cycle would require.

Quantitatively speaking, C23S/F285D holds an activity that is 1.5‐fold that of the wild‐type at its peak performance. Moreover, C23S/F285D is the most active variant across all temperatures at pH 7, and mostly on all occasions between pH 6.5–8.5. These constitute a clear improvement within standard biocatalysis conditions, which supports the employment of C23S/F285D as a good substitute for wild‐type *Cb*FDH in NADH recycling.

In order to assess the stability of the new enzymes, we measured the activity of each enzyme at different points in time after continuous incubation at 30 °C for 7 days. Table 1 summarizes the residual activities obtained from each *Cb*FDH variant after 24 h (day 2) and after 144 h of incubation (day 6). The relative residual activities show that even after 1 day, both C23S and C23S/F285D are more stable than the wild type, by 18% and 32%, respectively. Not only does the higher stability of these two variants become more pronounced after 5 days (up by 80% and 180%, respectively), but remarkably, the triple variant C23S/F285D/P286K displays 42% of its original activity (320% more than the wild type!). In conclusion, all of the variants vastly surpass the stability of the wild type after 5 days of incubation, with the triple variant displaying the best activity records after 1 week, rendering it ideal for work with multiple‐day reactions and slow processes.

**TABLE 1:** Residual activities of *Cb*FDH variants.

FDH variant	After 24 h		After 144 h	
	Relative residual activity	Relative to wt	Relative residual activity	Relative to wt
Wt	73%	n.a.	10%	n.a.
C23S	86%	+18%	18%	+80%
C23S/F285D	96%	+32%	28%	+180%
C23S/P286K	67%	−8%	25%	+150%
C23S/F285D/P286K	53%	−27%	42%	+320%

### 2.3 Structure–Activity Relationships

The fact that C23S/F285D has an improved activity and stability at standard conditions, and C23S/F285D/P286K has considerably higher stability at 30 °C for prolonged periods of time proves that both positions, 285 and 286, play a crucial role in the functionality of *Cb*FDH. In order to establish the relationship between the enzymatic structure and the activity response, a computational analysis was carried out. PyMol [65] and AlphaFold3 [75] were used to visualize and simulate each structure, and the conformational changes ensuing from each mutation.

**FIGURE 5:**
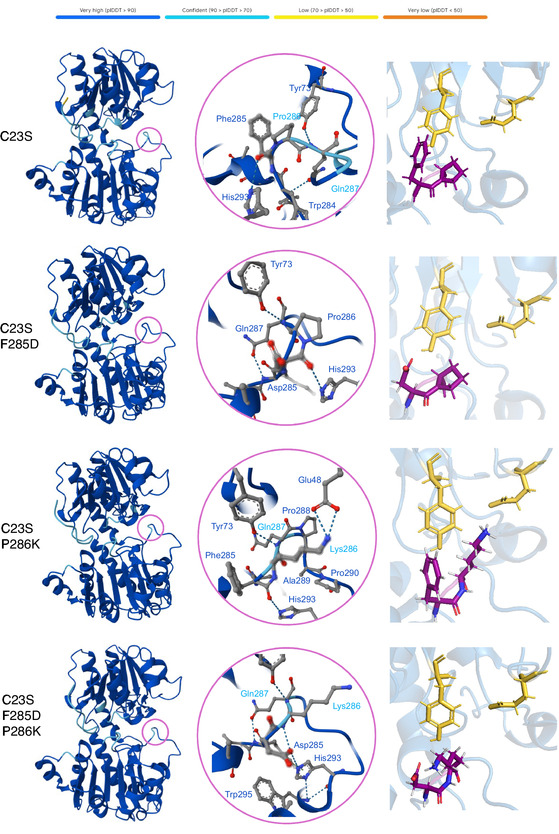
AlphaFold3 [75] (left and middle columns) and PyMol [65] (right column) visualizations of *Cb*FDH's (PDB:5DN9) [64] variants: C23S, C23S/F285D, C23S/P286K and C23S/F285D/P286K. On the AlphaFold3 structures, the folding is color‐coded according to the certainty of the simulation, as per the legend at the top. On the PyMol structures, Tyr73 and Glu48 are displayed in yellow, and Phe/Asp285 and Pro/Lys286 in purple. The overall position of the mutation within subunit A of *Cb*FDH is marked with the pink circle, which is then zoomed in in the middle column. As it can be seen, this location is right at the entrance of the catalytic channel.

The pH optimum shift, increased activity and variations in the temperature profile in C23S/F285D can be at least partly explained by looking at Figure 5. The second row, which corresponds to this variant, shows how the presence of aspartate in position 285 generates an interaction with glutamine 287, forcing them closer together than with the original Glu287‐Trp284 in C23S (Figure 5, first row). We also presume that the electrically charged aspartic acid would make stronger/easier hydrogen bonds and facilitate electron movement for catalysis compared to the uncharged benzene ring in phenylalanine.

In contrast, C23S/P286K has a clear negative effect on the enzyme, presenting the lowest activity of the set. We hypothesize that this loss of activity can be explained by the interactions—or lack thereof—of the newly instated lysine with tyrosine 73 and glutamic acid 48, which sit precisely at the entrance of the channel, acting as part of its “lid”. In all the other variants, where the activity is relatively high compared to C23S/P286K, there is a single interaction of Tyr 73 with Gln 287, which seems to be key for maintaining a good level of catalytic activity. However, in C23S/P286K, the long lysine creates an interaction with glutamate 48, which in effect displaces the rest of the fold in a way that we assume results in a narrower channel, making the entrance and exit of substrates and products complicated. The interaction with Glu 48 is not observed in any of the other enzymatic structures studied in this work. In fact, paradoxically, when phenylalanine 285 is also mutated to an aspartate, the latter forms a hydrogen bond with the lysine, causing it to bend sideways and out of the way of the tyrosine‐glutamic acid moiety, thus facilitating the entry and exit to the catalytic channel. This specific refolding of the lysine would explain why the variant C23S/F285D/P286K is considerably more active than C23S/P286K, but could only be observed when performing the mutation with PyMol (PDB: 5DN9, Wizard Protein Mutagenesis Tool) [64, 65], as represented in Figure 5, rightmost image on the last row. On the other hand, the simulation of the F285D/P286K on AlphaFold3 (Figure 5, last row, center image) shows no such bend in the lysine, and instead seems to indicate that the lysine is forced out of the way of the channel lid by interactions of the aspartate with His 293, which in turn interacts with Trp 295, creating a twist on the whole branch [64, 75]. Either way, the folding occurs in such a way that the Lys 286–Glu 48 interaction is nonexistent, and although the presence of the lysine clearly still affects the enzymatic activity to some degree, catalysis is still possible. It is important to remark that, unfortunately, in some instances, the AlphaFold simulations display amino acids P286 and Q287, which are crucial for this analysis, in a “confident” area (light blue) of folding, instead of the most desirable “very high confidence” (dark blue), which promises the most accurate results. Nevertheless, given the overall match between our AlphaFold3 and PyMol structures and the sound activity results, we still have a high degree of confidence in our conclusions.

Coincidentally, the distance between Pro/Lys 286 and Glu 48 was measured by uploading the AlphaFold3 simulated structures into PyMol (results in Table 2). These distances evidence the closure of the channel lid in C23S/P286K and its reopening in the triple variant.

**TABLE 2:** Measured distances with Glu 48.

FDH variant	Residues involved	Distance, Å
C23S	Glu48‐Pro286	7.4
C23S/F285D	Glu48‐Pro286	7.4
C23S/P286K	Glu48‐Lys286	2.8
C23S/F285D/P286K	Glu48‐Lys286	6.1

### 2.4 Synthetic Applications

The idea behind this article was to develop a formate dehydrogenase variant that overcomes the current intrinsic low activity of the wild‐type protein to unlock the true potential of the enzyme as an economic, convenient, and attractive alternative for NAD(P)H recycling. In order to assess C23S/F285D in a practical setting, we employed two benchmark biotransformation systems in which it can be used as an NADH recycling agent for highly specialized asymmetric reductase biocatalysis. We selected two reductase families that have considerably grown in popularity in recent years, ene‐reductases (ERED) [76, 78], and ketoreductases (KRED) [79, 81], and that are widely used in asymmetric synthesis. For practical reasons, we selected commercial enzymes from the respective Codexis screening kits, namely ERED 110 and KRED 459, as they were readily available on site and had been employed previously as synthetic tools by our group [82].

**SCHEME 1:**
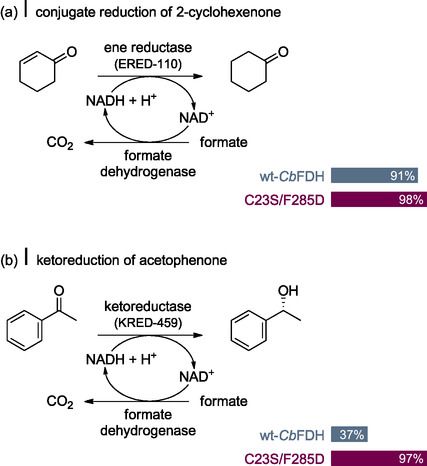
Benchmarking of the FDH‐based NAD^+^‐recycling system in the conjugate reduction of 2‐cyclohexenone and ketoreduction of acetophenone: yields with the wild‐type FDH are shown in slate blue, while yields with the C23S/F285D variant are shown in wine red.

Whilst C23S/F285D performs better in the two sets of reactions, it is true that the results with ERED110 do not pose as sharp a difference with the WT as expected. This difference does become more obvious with KRED459, in which C23S/F285D almost triples the yield of wt‐*Cb*FDH (Scheme 1).

## 3 Conclusions

The present work has been successful in rationally designing and developing three variants of *C. boidinii* formate dehydrogenase through site‐directed mutagenesis. In particular, C23S/F285D presents an improved activity (1.5‐fold that of the wild type at its peak performance, and 1.8‐fold at pH 7 and 40 °C) and friendlier pH and temperature profiles to facilitate NADH recycling. The cofactor recycling was put into practice with an ERED and a KRED, and in both instances, FDH‐C23S/F285D outperforms recombinant wild‐type *Cb*FDH. In studying two residues close to the “lid” in the catalytic channel, F285 and P286, we have established that these two positions play a crucial effect toward enzymatic activity. The exploration of mutations *Cb*FDH‐C23S/F285D, *Cb*FDH‐C23S/P286K, and *Cb*FDH‐C23S/F285D/P286K in both PyMol and AlphaFold3 has unearthed that both tyrosine 73 and glutamate 48 are directly involved in the catalytic process by “opening” or “closing” the channel through interactions with positions 285 and 286. C23S/F285D shows that the aspartate is able to create a stronger network of connections with its surroundings, including Tyr73, which results in tighter folding and a wider entrance of the substrate channel, resulting, as the research proves, in better enzymatic activity. On the other hand, C23S/P286K experiences a considerable loss of catalytic power, which the observation of the structural models helps in rationalize: lysine does not have any particularly strong interactions with phenylalanine 285, which leads to its spreading upward, forming new interactions with glutamate 48 and, in effect, rendering the substrate channel much narrower, and in essence challenging access to the channel. The triple variant C23S/F285D/P286K displays an acceptable level of activity in spite of carrying lysine 286. Both our PyMol model and our AlphaFold3 simulation support this result, although in slightly different manners. While the PyMol analysis suggests that lysine 286 has a strong interaction with aspartate 285, resulting in a clear folding of the lysine that opens the channel by avoiding the connection with Glu 48, AlphaFold3 suggests that the opening of the channel is the result of the stronger interactions of aspartate with its surroundings, effectively pulling the lysine far from glutamate 48. In any case, both theories are plausible and reasonable, and both are in accordance with the experimental results.

## Funding

This study was supported by Research Council of Finland (Grant 329510), and European Research Council (Grant 865885).

## Conflicts of Interest

The authors declare no conflicts of interest.

## Supporting information

Supplementary Material

## Data Availability

The data that supports the findings of this study are available in the supporting information of this article.
