# Supplementary material for: Improving Activity and Stability of Candida boidinii Formate Dehydrogenase Through Rational Active Site Engineering
Source: Chembiochem. 2026 Aug 14;27(15):e70497. doi: 10.1002/cbic.70497 (PMC13475586; doi:10.1002/cbic.70497)
Supplement: Supplementary file 1 [file CBIC-27-e70497-s001.pdf]

Improving Activity and Stability of *Candida boidinii* Formate Dehydrogenase Through Rational Active Site Engineering

Marisa Bickmann,[b] Andrea Rodil,[a]* and Jan Deska*[a]

S1. General Methods

All glassware and plasticware used for biological purposes was autoclaved in advanced and used inside a laminar flow cabinet (Kojair). Media and buffers, when self-made, were also sterilised by autoclaving. These procedures were carried out in a Laboklav, SHPSteriltechnik AG, and using the pre-set programmes. Otherwise, materials were provided and already sterilised by the media kitchen of the Helsinki Institute of Life Sciences of the University of Helsinki (Viikki Campus).

Commercially available CbFDH enzyme was purchased from Merck KGaA (Darmstadt). The synthetic, codon-optimised genes for WT-FDH and FDH_C23S_F285D_P286K were purchased from Eurofins Genomics (Germany). Both were ordered with BamHI at 5' restriction site, and HindIII at 3' restriction site. The genes WT-FDH and its variants by site-directed mutagenesis, were inserted in a pET-28a(+) vector which was readily available in the lab, and transformed into *E. coli* DH5α (ThermoFisher) for cloning and into *E. coli* BL21 DE3 (ThermoFisher) for overexpression. Variants C23S, C23S_F285D and C23S_P286K, were obtained by site-directed mutagenesis adapting the procedure described by Edelheit (2009). The polymerase used was PrimeSTAR™ HS Premix by Takara, or Phusion™ High-Fidelity DNA Polymerase by ThermoFisher and the specific primers were ordered from Eurofins genomics. PCRs were performed in an Eppendorf Mastercycler X50a. FDH_C23S_F285D_P286K was delivered directly in pET28a-(+) and were transformed into *E. coli* BL21 DE3 directly.

Isolation of plasmid DNA from bacterial cultures was done with a GeneJET Plasmid Miniprep Kit, whilst purification of DNA from agarose gels was done with a GeneJET Gel Extraction Kit. Both were used according to their provided protocols, and both were purchased from ThermoFisher (Waltham).

DNA sequencing to confirm desired mutations was done by Eurofins Genomics via Sanger sequencing on ABI 3730XL devices (Applied Biosystems).

Incubations of 2 mL or less were carried out in a Digital Heatblock by Benchmark, while larger scale incubations were done in ThermoScientific incubators. Cell lysis was performed in a UP400S ultrasonic processor by Hielscher Ultrasonics (Teltow), set to 50% cycle and 35% amplitude. Centrifugation was carried out in a Multifuge X Pro Series centrifuge by ThermoScientific and rotors of the FiberLite series.

Sodium dodecylsulfate polyacrylamide gel electrophoresis (SDS-PAGE) was run using either self-made gels, or commercially available Mini-PROTEAN TGX Precast Gels by BioRad. Gels were run in a Bio-Rad Mini-PROTEAN tetra system chamber with tris-glycine running buffer. Samples were prepared using Novex™ Tris-Glycine SDS Sample Buffer (ThermoFisher) and size was referenced to PageRuler™ Prestained Protein Ladder. Gels were visualised with ReadyBlue™ Protein Gel Stain (Merck).

Agarose gels were self-made in the lab using UltraPure™ Agarose (Invitrogen), following their standard protocol in TAE buffer. 50X TAE buffer for Electrophoresis was purchased from ThermoScientific. Gels were cast and ran in Bio-Rad equipment. Agarose gel electrophoresis was used for confirmation and/or isolation of restriction digest, the subcloning and PCR amplification. For preparative gel electrophoresis, 18 μL sample were mixed with 3 μL of TriTrack DNA Loading Dye (6x). For analytical gels 1 μL sample, 1 μL loading dye and 4 μL dH_2O were used. As a size standard 1 μL of the GeneRuler DNA Ladder Mix (Thermo Fisher) was mixed with 1 μL TriTrack and 4 μL dH_2O and fully loaded onto the gel. The gels were run at 100 V for 40 min, rinsed with dH_2O and then stained in a 1:10000 solution of 1x TAE and EmeralDye (Takara) for 20 min under constant agitation.

Visualisation and recording of acrylamide and agarose gels was carried out in a gel imager Azure200 by Azure Biosystems.

All biological samples were flash-frozen and stored at -80 °C, unless otherwise stated. DNA, antibiotic stocks and PCR materials were stored at -20 °C.

Reagents that were not readily available in the lab were obtained from ThermoFisher Scientific, Sigma Aldrich or BDL Pharm, and were used without any further purification. “Room temperature” refers to conditions between 18-25 °C.

Analytical thin layer chromatography (TLC) was carried out on an aluminium-backed silica gel with 200 μm layer thickness (VWR). Visualisation of compounds was achieved under UV light (254 nm), or by staining with *p*-anisaldehyde-sulfuric acid solution followed by thermal development.

GCMS analysis was performed in a Bruker Scion TQ, according to the following programme:

Table S1. GCMS conditions

Temperature (°C)	Rate (C/min)	Hold (min)	Total (min)
60	-	2.00	2.00
165	15.0	5.00	14.0
200	25.0	5.00	20.40
300	20.0	2.00	27.40

S2. Genetic Engineering Methods

S2.1 Gene Sequences

The gene for FDH WT (wild type) was ordered from Eurofins Genomics, using the sequence published by Guo et al. (**2016**), deposited in PDB with the code 5DNA.[1]

GGA TCC ATG AAG ATT GTT CTG GTG CTC TAT GAT GCC GGG AAA CAT GCC GCT GAT GAA GAG AAA CTG TAT GGC TGC ACG GAA AAC AAA CTG GGC ATT GCC AAT TGG CTC AAA GAT CAG GGG CAT GAG CTG ATT ACC ACG TCG GAT AAA GAG GGC GGT AAT AGC GTG CTG GAC CAG CAC ATT CCG GAC GCA GAC ATT ATC ATC ACT ACC CCG TTT CAT CCG GCG TAC ATC ACG AAA GAA CGC ATT GAT AAG GCC AAA AAG CTG AAA TTG GTG GTT GTG GCT GGT GTA GGC AGC GAT CAC ATC GAC CTT GAT TAC ATC AAC CAG ACC GGC AAA AAG ATT AGC GTC CTG GAA GTT ACC GGC AGT AAT GTG GTG AGT GTT GCA GAA CAT GTC CTG ATG ACC ATG CTG GTT CTT GTC CGC AAC TTT GTA CCT GCA CAT GAA CAG ATC ATC AAC CAC GAT TGG GAA GTA GCC GCG ATT GCG AAA GAC GCG TAT GAC ATC GAA GGC AAA ACA ATC GCG ACT ATT GGG GCT GGT CGC ATT GGG TAT CGC GTA CTT GAA CGC TTA GTC CCC TTT AAC CCG AAA GAG TTA CTG TAC TAT GAT TAC CAG GCG TTA CCG AAA GAT GCG GAA GAA AAA GTC GGA GCG CGT CGT GTG GAA AAC ATT GAA GAG CTG GTT GCG CAA GCG GAT ATT GTG ACG ATT AAT GCG CCA CTG CAT GCG GGC ACA AAG GGT CTG ATC AAC AAA GAA CTG CTG TCG AAA TTC AAA AAA GGT GCT TGG TTG GTG AAT ACA GCA CGT GGA GCC ATT TGT GTC GCT GAA GAT GTG GCA GCA GCC TTG GAA TCA GGC CAG TTA CGT GGC TAT GGT GGG GAT GTT TGG TTT CCG CAA CCA GCC CCG AAA GAC CAT CCA TGG CGC GAT ATG CGC AAC AAA TAC GGT GCT GGT AAT GCC ATG ACT CCC CAC TAC TCT GGT ACC ACG CTC GAT GCC CAA ACC CGG TAT GCA GAA GGT ACC AAG AAC ATC TTG GAG TCC TTT TTC ACG GGC AAA TTC GAC TAC CGT CCT CAG GAT ATT ATT CTG CTG AAT GGC GAG TAT ATC ACC AAA GCG TAT GGA AAG CAC GAC AAA AAA AAG CTT

The gene for FDH C23S_F285D_P286K was ordered from Eurofins genomics, using the sequence published by Guo et al (2016) as a base,[1] and adding the three modifications in positions 23 (cysteine for serine), 285 (phenylalanine for aspartate) and 286 (proline for lysine).

GGA TCC ATG AAA ATT GTG TTA GTC CTT TAT GAC GCA GGC AAA CAC GCG GCG GAT GAG GAG AAA CTC TAT GGG TCC ACC GAG AAC AAG TTG GGT ATT GCC AAT TGG CTG AAA GAT CAG GGT CAC GAA CTG ATC ACG ACC TCA GAT AAG GAA GGT GGG AAT AGC GTA CTG GAT CAG CAT ATC CCG GAT GCG GAC ATC ATC ATT ACC ACA CCG TTT CAT CCG GCG TAC ATT ACC AAA GAA CGC ATC GAT AAA GCG AAA AAA CTG AAA CTG GTT GTT GTG GCT GGT GTG GGC TCG GAT CAC ATT GAC TTG GAC TAC ATC AAC CAG ACA GGC AAA AAG ATC AGT GTT CTG GAA GTG ACC GGT AGC AAT GTG GTG TCT GTG GCG GAA CAT GTC CTG ATG ACC ATG CTG GTC TTG GTT CGC AAC TTT GTT CCA GCG CAT GAA CAG ATT ATT AAC CAC GAC TGG GAA GTT GCG GCC ATT GCG AAA GAT GCC TAC GAT ATC GAA GGC AAA ACG ATT GCC ACT ATT GGA GCA GGC CGC ATT GGC TAT CGT GTG CTG GAA CGC CTG GTA CCG TTT AAC CCC AAA GAA CTC CTC TAC TAC GAC TAT CAG GCT TTA CCC AAA GAT GCA GAG GAA AAA GTC GGC GCT CGT CGG GTA GAG AAC ATC GAG GAA CTT GTA GCA CAA GCC GAC ATT GTG ACG ATT AAT GCA CCG TTA CAT GCG GGA ACT AAA GGC CTG ATC AAC AAA GAG CTG CTG AGT AAG TTC AAG AAA GGC GCG TGG TTG GTG AAT ACG GCC CGT GGA GCC ATT TGC GTC GCT GAA GAT GTT GCC GCA GCT CTT GAA AGC GGT CAA CTG CGT GGT TAT GGT GGC GAT GTC TGG GAT AAA CAG CCA GCT CCT AAA GAC CAT CCG TGG CGT GAT ATG CGC AAT AAG TAC GGT GCA GGG AAC GCG ATG ACC CCT CAC TAT TCC GGG ACA ACG TTA GAT GCC CAG ACT CGC TAT GCG GAA GGT ACG AAG AAC ATT CTG GAA TCG TTC TTT ACC GGG AAA TTC GAT TAT CGC CCG CAA GAC ATC ATT CTG CTG AAT GGC GAA TAC ATC ACC AAA GCC TAT GGC AAA CAT GAC AAA AAA AAG CTT

S2.2 Plasmid preparation

The FDH WT gene was delivered as a lyophilised residue in the commercial pEX-A258 plasmid (**Fig. S1**).

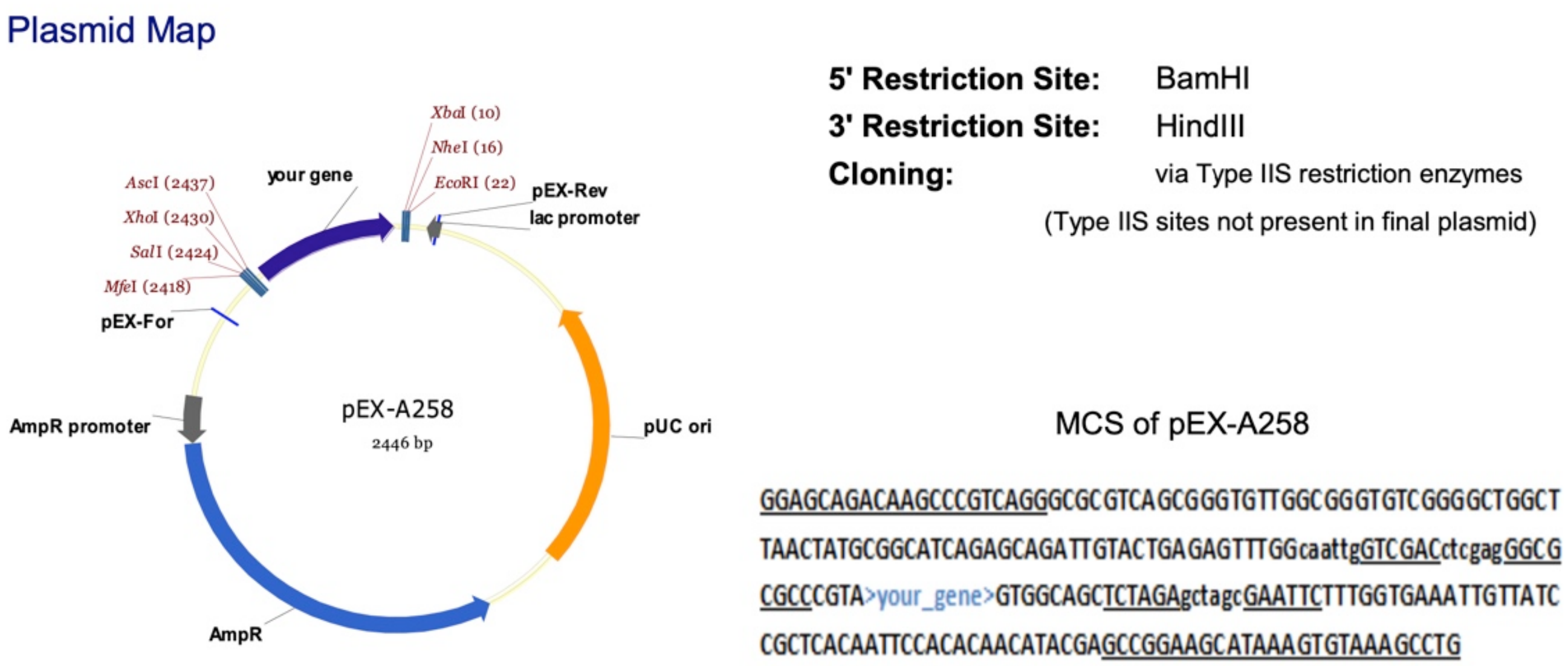

Figure S1. Vector map of FDH gene in pEX-A58, as delivered from Eurofins Genomics.

In order to subclone the gene into the pET28a-(+) destination vector, a digest with the restriction endonucleases BamHI and HindIII was performed on both plasmids, as shown in Table S2.

Table S2. Set up for the reactions to transfer the gene from pEX-A258 to pET28a-(+)

Component	Volume	Final Concentration
DNA	-	50 ng/µL
BamHI buffer (10x)	2 µL	1x
BamHI [10 U/µL]	1 µL	0.5 U/µL
HindIII [10 U/µL]	2 µL	1 U/µL
dH_2O	To 20 µL	-

The reaction mixtures were incubated for 4 h at room temperature. A quantitative agarose gel was run with the digestion mixtures and bands of the insert (Cb*fdh*) and backbone (pET28a-(+)) were purified from the gel for ligation. A T4 DNA ligase from Thermo Fisher was used for the ligation of digested DNA fragments. For a ligation set up 0.3-30 fmol of the backbone and 0.9-90 fmol of the backbone was used, whereas the ratio of insert and backbone was 3:1. To calculate the volume of the DNA needed formula 2 was used:

(S1)
$$V[\mu L] = n[\mathrm{pmol}]\,\frac{0.66\left[\frac{\mathrm{ng}}{\mathrm{pmol}}\right]\mathrm{bp}}{c_{DNA}\left[\frac{\mathrm{ng}}{\mu L}\right]}$$

n: Amount of DNA for ligation

0.66 ng/pmol:	Average molecular weight of one base pair
bp:	Size of DNA fragment
c_{DNA}:	Concentration of DNA

For a ligation insert, backbone, 1x T4 DNA ligase buffer, 1 U T4 DNA ligase and dH_2O to 20 µL were mixed and incubated for 20 min at room temperature. 1 µL of the ligation mixture was directly used for transformation of *E. coli* Dh5α.

S2.3 Polymerase Chain Reaction (PCR) and Site-directed mutagenesis

Polymerase chain reactions were used to introduce site-directed mutations into the CbFDH gene. The primers used are shown in Table S3.

Table S3. Primers for site-directed mutagenesis as purchased from Eurofins Genomics.

Primer	Mutation	Sequence
MB01-fw	F285D	CTATGGTGGGGATGTTTGGGATCCGCAACCAGCCCCGAAAG
MB01-rv	F285D	CTTTCGGGGCTGGTTGCGGATCCCAAACATCCCCACCATAG
MB02-fw-1	P286K	GTGGGGATGTTTGGTTTAAACAACCAGCCCCGAAAG
MB02-rv-1	P286K	CTTTCGGGGCTGGTTGTTTAAACCAAACATCCCCAC
MB03-fw-1	C23S	GAGAAACTGTATGGCAGCACGGAAAACAAAC
MB03-rv-1	C23S	GTTTGTTTTCCGTGCTGCCATACAGTTTCTC

For this, the Single-Primer Reactions in Parallel (SPRINP) PCR method (Edelheit et al., **2009**)[2] was modified as follows.

Table S4. Reaction set up for the PCR. Individual components and final concentrations are shown.

Component	Final concentration
Primer (forward <u>or</u> reverse):	40 pmol
dNTPs (desoxy nucleoside triphosphate)	200 µL each
Phusion buffer	1x
Phusion® High-Fidelity DNA Polymerase	0.02 U/L
Template DNA	2.5 ng/µL
dH_2O	To 20 µL

OR

Component	Final concentration
Primer (forward or reverse):	40 pmol
PrimeStar™ Premix	10 μL
Template DNA	2.5 ng/μL
dH_2O	To 20 μL

Table S5. Site-directed mutagenesis parallel reactions thermal protocol

Step	Temperature [°C]	Time [min:sec]	
1	98	00:10	
2	98	00:10	30 cycles
3	55	00:15	30 cycles
4	72	01:00	30 cycles
5	72	01:00	
6	4	Hold	

After the PCR, the reactions were combined (40 μL total), gently mixed, and heated in a thermocycler following this protocol (table S6):

Table S6. Temperature ramp-down protocol for the combined PCR reactions

Step	Temperature [°C]	Time [min:sec]
1	95	05:00
2	90	01:00
3	80	00:30
4	70	00:30
5	60	00:30
6	50	00:30
7	40	00:30
8	37	Hold

To digest any methylated parental DNA from the PCR reactions, 1 µL (10 U) of DpnI was added to the combined PCR reactions after the heating protocol. The reactions were mixed gently and then incubated at 37 °C overnight. After the digest, 1 µL of the mixture was directly used for transformation.

S2.4 Transformation into *E. coli*

Chemically competent cells of the *E. coli* strains DH5α and BL21(D3) were purchased from *Thermo Fisher* (Waltham), aliquoted to 100 µL and stored at -80 °C. Prior to a transformation, aliquots were thawed on ice for 10-30 min. DNA was added and the cells were incubated on ice for an additional 30 min. The cells were then subjected to heat shock at 42 °C for 30 s and immediately put on ice for 2 min. LB medium (preheated to 42 °C, 700-950 µL) was added and the cells were incubated at 37 °C for one hour at 180 rpm. After incubation, the samples were centrifuged at 13000 rpm, the supernatants discarded, and the pellets resuspended in the residual medium. The whole batch was plated on LB agar plates containing the appropriate antibiotic and incubated overnight at 37 °C.

S3. Protein production and purification

S3.1 Cell growth, glycerol stocks and protein production

Precultures were inoculated by addition of a bacterial colony (picked from the agar plate with a sterile pipette tip) into 5 mL of LB containing either 100 µg/mL ampicillin or kanamycin, as required. The precultures were incubated at 37 °C and 180 rpm overnight.

For protein production, expression cultures were prepared. In a 500 mL Erlenmeyer flask, 200 mL of LB medium with 100 µg/mL kanamycin was inoculated with 1 mL of the preculture and incubated at 37 °C and 180 rpm until OD_{600} = 0.6-0.8 was reached. The cells were put on ice for 10 min and gene expression was induced by addition of IPTG to a final concentration of 0.5 mM. The cells were incubated at 25 °C and 180 rpm for 24 h. After incubation the cells were harvested by centrifugation at 4 °C and 5000 rpm for 25 min with a *Fiberlite*™ F14-6x250 LE rotor. The supernatant was discarded, and the cell pellet was transferred to a sterile falcon tube, weighted, and stored at -80 °C for later use.

Prior to IPTG addition, glycerol stocks of the bacterial cultures were made. For this, 500 µL of bacterial overnight cultures were gently mixed with 500 µL 50 % (*w/w*) sterile glycerol solution, flash frozen in liquid nitrogen and stored at -80 °C.

S3.2 Cell disruption, enzyme purification and desalting

For protein purification, cell pellets from the expression cultures were thawed on ice or used fresh to be disrupted via sonification. Beforehand, one tablet of *cOmplete™ Mini EDTA-free Protease Inhibitor Cocktail* (*Roche*) was dissolved in 10 mL of lysis buffer (10 mM KP_i, 150 mM NaCl, 10 mM imidazole, pH 7.8). 1 g of frozen cell pellet was resuspended in 4 mL of the buffer in a 50 mL falcon tube using a vortex shaker. The resuspended cells were sonicated on ice using a UP400S ultrasonic processor by Hielscher Ultrasonics (Teltow) set to 50 % cycle and an amplitude of 35 %. Sonication was done for at least 5 min in 30 second pulses with breaks of 30 s. After sonication

the disrupted cell mixture had cleared noticeably in comparison to the resuspended cells. The disrupted cells were centrifuged for 40 min at 4 °C and 1200 rpm. The lysate was transferred to a fresh falcon tube and kept on ice for further purification of the protein.

The produced CbFDH was purified using immobilized metal affinity chromatography with nickel nitrilotriacetic acid (Ni-NTA) resin. Empty columns were filled with 3 mL Ni-NTA resin and equilibrated with 5 colum volumes (CV) of lysis buffer (10 mM KP_i, pH 7.8, 20 mM imidazole, 150 mM NaCl). Lysate was passed through the column, the flowthrough was collected and then passed through the column again. The column was washed with wash buffer (10 mM KP_i, pH 7.8, 70 mM imidazole, 150 mM NaCl) in two steps, using first 4 CV and then 2 CV. Elution of the CbFDH was done in three steps. For each step, a fresh 15 mL falcon tube was placed underneath the column. 1 CV of elution buffer (10 mM KP_i, pH 7.8, 500 mM imidazole, 150 mM NaCl) was used for elution 1, 2 CV were used for elution 2 and 1 CV was used for elution 3.

SDS-PAGE (sodium dodecyl sulfate–polyacrylamide gel electrophoresis) was used to monitor the purification of the FDHs. Samples were prepared as follows: For disrupted cells 10 µL sample were mixed with 45 µL dH_2O and 15 µL *Novex™ Tris-Glycine SDS Sample Buffer* (*Thermo Fisher*). For the crude lysate, 5 µL of sample were mixed with 5 µL of dH_2O and 5 µL of sample buffer; and every other sample of the purification were prepared with 10 µL sample and 5 µL sample buffer. The SDS-PAGE samples were thoroughly mixed and then heated at 85 °C for 5 min.

To run the SDS-PAGE 10 µL of each sample was loaded onto a precast gel. As a size reference 5 µL of *PageRuler™ Prestained Protein Ladder* were used. 1x tris-glycine running buffer was prepared in advanced from a 10x concentrated buffer solution, which was provided by the media kitchen of the *Helsinki Institute of Life Sciences* (*University of Helsinki*). The electrophoresis was run for 50 min at 150 V. Gels were subsequently stained with *ReadyBlue™ Protein Gel Stain* (*Merck*) for 20 min under constant agitation. To de-stain the background, the gel was then incubated in dH_2O while shaking for 30 min. To help the de-staining process, a lint-free tissue was added. Pictures of the gels were taken using the *Azure 200* gel imager by *Azure Biosystems* (Dublin, California).

To get rid of the high imidazole concentrations after the IMAC, purified protein samples were desalted using *PD-10 desalting columns* (*GE Healthcare Technologies*). The tip of a column was cut off and the storage liquid was drained. The column was then equilibrated with 5 x 5 mL 100 mM KP_i buffer (pH 7.5). 2.5 mL of purified protein solution were loaded onto the column and the flowthrough was discarded. An empty 15 mL falcon tube was placed under the column and the protein was eluted with 3.5 mL of KP_i buffer (100 mM, pH 7.5).

S4. Enzymatic Assays

S4.1 Bradford assay for the determination of protein concentration

In addition to the use of a DeNovix spectrophotometer for protein concentration, a Bradford assay was used to determine the specific protein concentration in samples. For each assay, a new calibration curve using bovine serum albumin (BSA) in KP_i buffer (100 mM, pH 7.5) with known concentrations (150, 125, 100, 75, 50, 25, 10, 0 µg/mL) was measured. The measurements were done using a 96 well microtiter plate. 20 µL sample were mixed with 180 µL of *Bradford* reagent (*Thermo Fisher*) and incubated at room temperature for 15 min. Samples were diluted to 1:10, 1:100 and 1:1000 and measured in triplicates. The absorption at 595 nm was measured with a plate photometer to determine the protein concentration of the samples. The measurement points of the BSA solutions were plotted against the concentration and then used to calculate a linear regression curve (y=mx+b) with a coefficient of determination >0.98. The regression curve was then used to calculate the protein concentration of the samples using formula S2.

(S2)
$$c_{protein}\left[\frac{mg}{mL}\right] = \frac{\left(A_{595} - b_{reg}\right)}{m_{reg} * D * 1000}$$

A_{595}: Absorption at 595 nm
b_{reg}: Y intercept of regression curve
m_{reg}: Slope of regression curve
D: Dilution factor

The mean value was calculated from the protein concentrations of the triplicates, whereas values with too large a deviation were not taken into account. Subsequently, the standard deviations (σ) of the mean values were calculated (formula S3).

(S3)
$$\sigma = \sqrt{\frac{\Sigma(x_i - \bar{x})^2}{(n-1)}}$$

$\bar{x}$: Mean value
n: Sample size

S4.2 Enzyme activity assays

The enzyme activity of the formate dehydrogenases was determined by photometrically following the conversion from NAD^+ to NADH during the oxidation of formate to CO_2. Over a period of 15 min, the formation of NADH was measured by taking absorption values at 340 nm; the temperature was set at 30 °C, and the absorption of NADH

measured in 30s intervals, with double orbital shaking inbetween measurements at 807 cycles per minute. The assay set up is summarized in Table S7.

Table S7: Activity assay set up.

Component	Volume	Final concentration
100 mM KP_i pH 7.5	130 µL	
1 M NaCOOH	40 µL	200 mM
10 mM NAD^+	20 µL	1 mM
Enzyme	10 µL	

To calculate the enzymatic activity, a calibration curve with different NAD^+ concentrations was plotted. 20 µL of NAD^+ dilutions in dH_2O were mixed with 180 µL KP_i buffer (100 mM, pH 7.5) to final NAD^+ concentrations of 0, 0.02, 0.03, 0.03, 0.05, 0.07, 0.1, 0.12, 0.15, 0.17, 0.2, 0.25, 0.3, 0.35, 0.4 mM. Absorption at 340 nm at 30 °C was measured in triplicates and the resulting regression curve was calculated (figure S2).

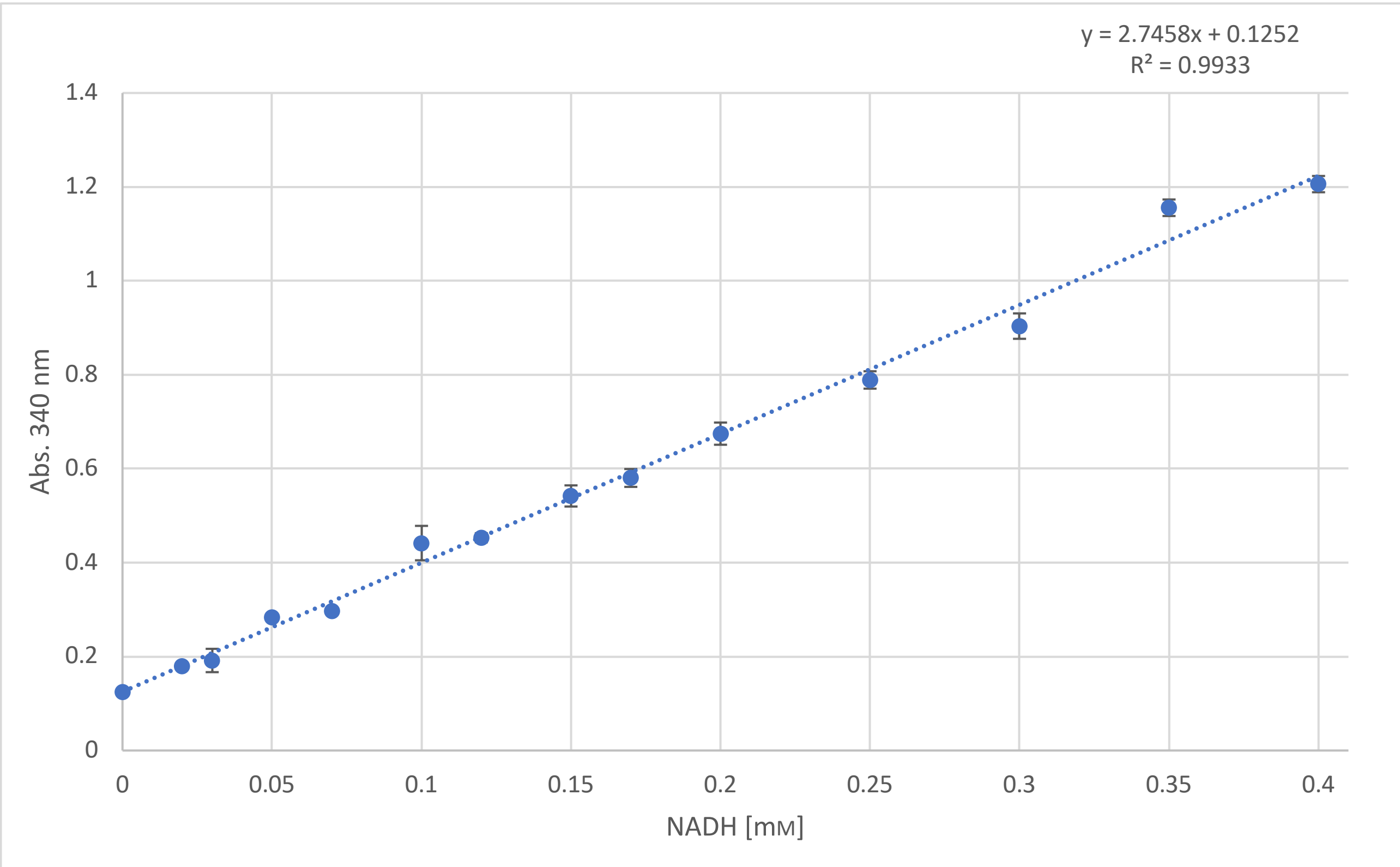

Figure S2. Measurements of the NADH calibration with the resulting regression curve.

To execute the activity assay, enzyme solution was pipetted into a well of a 96 well plate. To start the reaction the other components were added at the same time and the absorbance was immediately measured. Activity of the sample was calculated with the regression curve in Figure S2. Using the slope of the regression curve and the

slope of the linear range of the measurement the activity was calculated (formula S4). Based on the activity, the volumetric activity v (formula S5) and specific activity s (formula S6) were then calculated.

(S4) $$a\,[U] = \frac{m_{sample} * V_{well} * 60 * 1000}{m_{reg}}$$

(S5) $$v\,[U * mL^{-1}] = \frac{a * 1000}{V_{enzyme} * D}$$

(S6) $$s\,[U * mg^{-1}] = \frac{v}{c_{protein}[mg * mL^{-1}]}$$

m_{sample}:	Measured linear slope dA_{340}/dt
m_{reg}:	Slope of regression curve (see figure 30)
V_{well}:	Volume per well, 200 µL
V_{enzyme}:	Volume of the enzyme solution, 10 µL
D:	Dilution factor
$c_{protein}$:	Protein concentration determined via *Bradford* assay

S4.3 Stability assays

The stability of the formate dehydrogenases was determined by photometrically following the conversion from NAD^+ to NADH during the oxidation of formate to CO_2 over a period of 7 days. The assays were continuously incubated at 30 °C, with no shaking, and the formation of NADH was measured by taking daily absorption values at 340 nm; at 30 °C, and the absorption of NADH measured in 30s intervals, with double orbital shaking inbetween measurements at 807 cycles per minute. The assay set up is summarized in table S8. Prior to each daily measurement, 4 µL of NaCOOH (1M stock) and 1 µL of NAD^+ (10 mM stock would be added).

Table S8: Stability assay set up.

Component	Volume	Notes
100 mM KP_i pH 7.5	130 µL	
1 M NaCOOH	4 µL	To be added daily
10 mM NAD^+	1 µL	To be added daily
Enzyme	10 µL	

The activity was then calculated as previously described.

S4.4 Kinetic experiments

To determine the kinetic parameters of formate and NAD^+ of CbFDH wild type and all the newly produced variants, the activity of the enzyme was measured using the absorbance of NADH at 340 nm in a set of variable formate and NAD^+ concentrations, respectively. Michalis-Menten values (Km) and Vmax were calculated for each case (Table S9).

Table S9. Michaelis-Menten values for formate and NAD^+ for each variant

	Km, formate (mM)	Vmax, Formate (U)	Km, NAD^+ (mM)	Vmax, NAD^+ (U)
**Wild Type**	37.6±3.35	0.0077±1.40	0.35±0.03	0.0068±1.06E-4
**C23S**	16.2±1.43	0.0099±1.7E-4	1.24±0.13	0.020±8.42E-4
**C23S/F285D**	37.6±3.35	0.026±3.88E-4	1.76±0.15	0.0419±9.47E-4
**C23S/P286K**	20.5±1.62	0.00671±9.77E-5	0.68±0.0531	0.0131±2.81E-4
**C23S/F285D/ P286K**	8.81±1.60	0.030±9.98E-4	0.73±0.14	0.0203±8.34E-4

To determine the kinetic parameters of NAD^+ and formate, the above activity assay was used. For the measurement of the kinetic values of NAD^+, the final NAD^+ concentrations changed, while a formate concentration was kept at 40 mM. For the formate kinetic measurements, the formate concentrations changed, while NAD^+ was maintained at 10 mM. The final concentrations used for each substrate are listed in table S9.

Table S10: Used formate (**12**) and NAD^+ concentrations of the kinetic measurements.

**Formate** [mM]:	600, 550, 500, 470, 430, 400, 350, 300, 270, 230, 200, 150, 100, 60, 40, 20, 14, 12, 10, 6, 5, 4, 3, 2
**NAD^+** [mM]:	15, 12, 10, 8, 6, 3, 1, 0.8, 0.6, 0.2, 0.08

For the kinetic measurements all components except for the enzyme were pipetted into the wells of a 96 well microtiter plate and mixed thoroughly. The enzyme solution was added last, and the measurement was started immediately. The activity was calculated as previously described.

S4.4.1 Recombinant wild type CbFDH

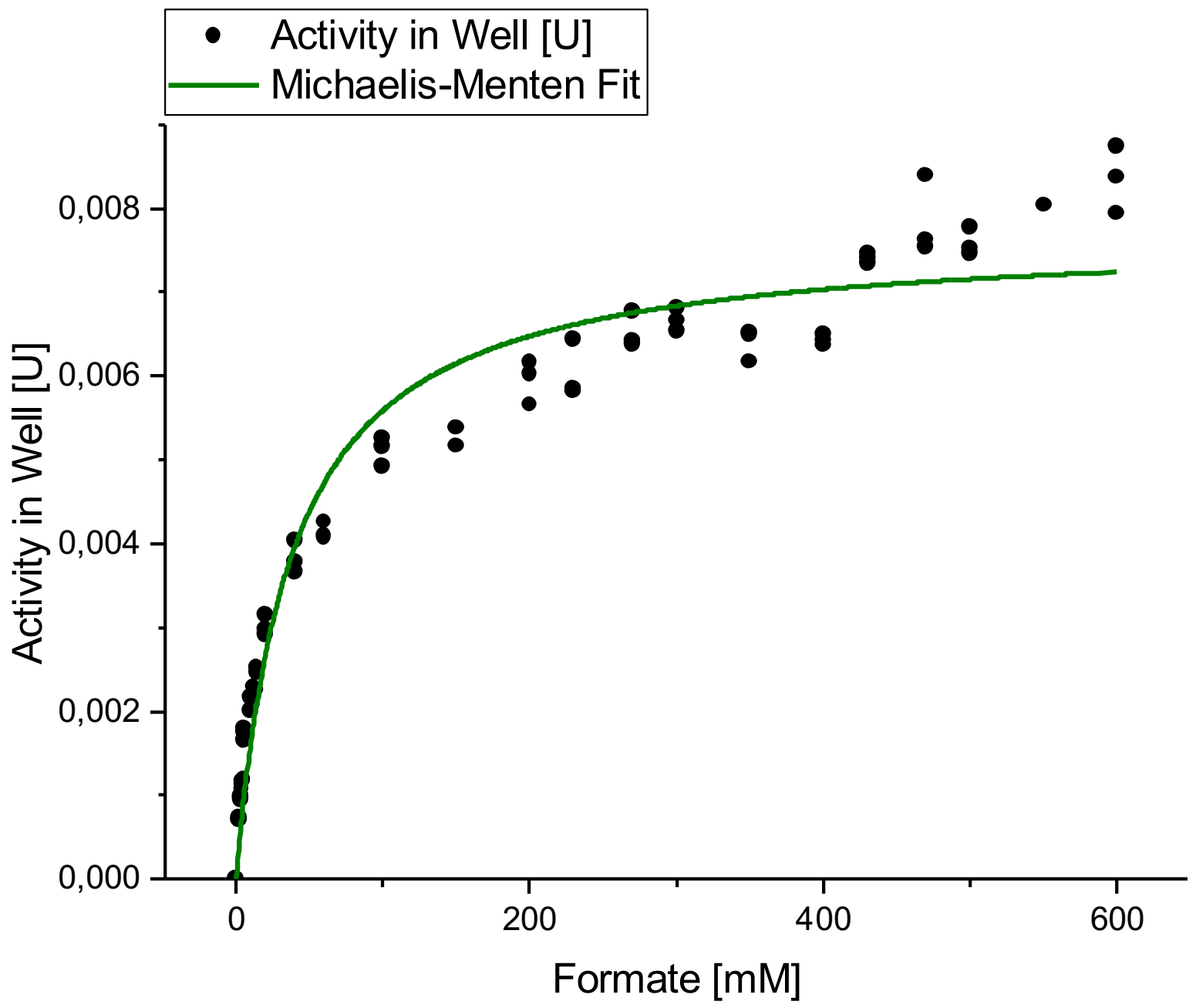

Figure S3. Formate kinetics for wild type CbFDH. The activity in the wells (U) was plotted against formate concentration (mM). Data points were fitted using the Michaelis-Menten formula on OriginPro software.

Using OriginPro software, the maximal velocity V_{max} was determined to be 0.0077 ± 1.40E-4 U and K_M was calculated to be 37.6±3.35 mM, with an R^2 value of 0.96 for the plot.

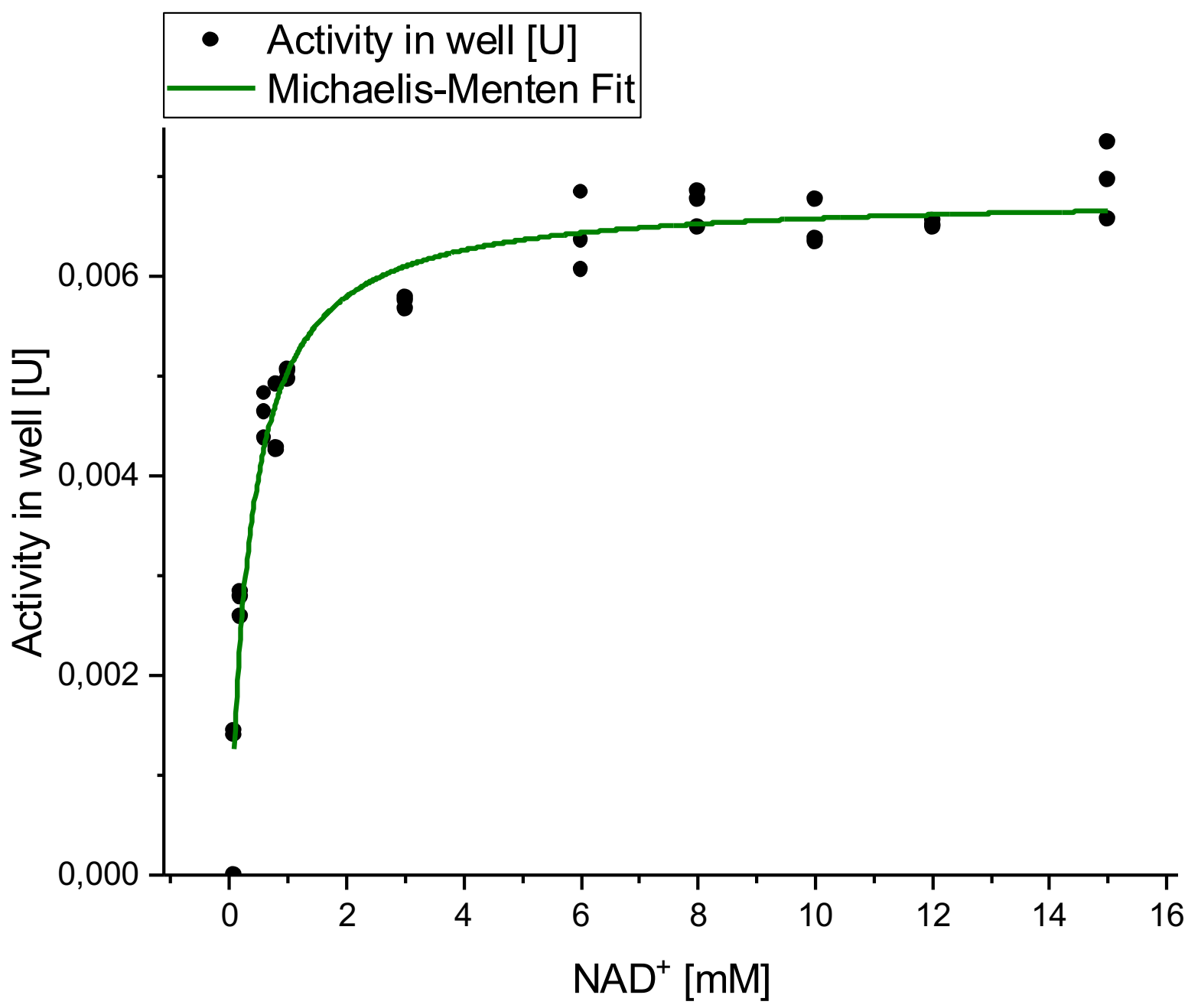

Figure S4. NAD^+ kinetics for wild type CbFDH, plotted using OriginPro.

Maximal velocity V_{max} was determined to be 0.0068 ± 1.06E-4 U and K_M was calculated to be 0.35±0.03 mM, with an R^2 value of 0.96 for the plot.

S4.4.2 CbFDH-C23S

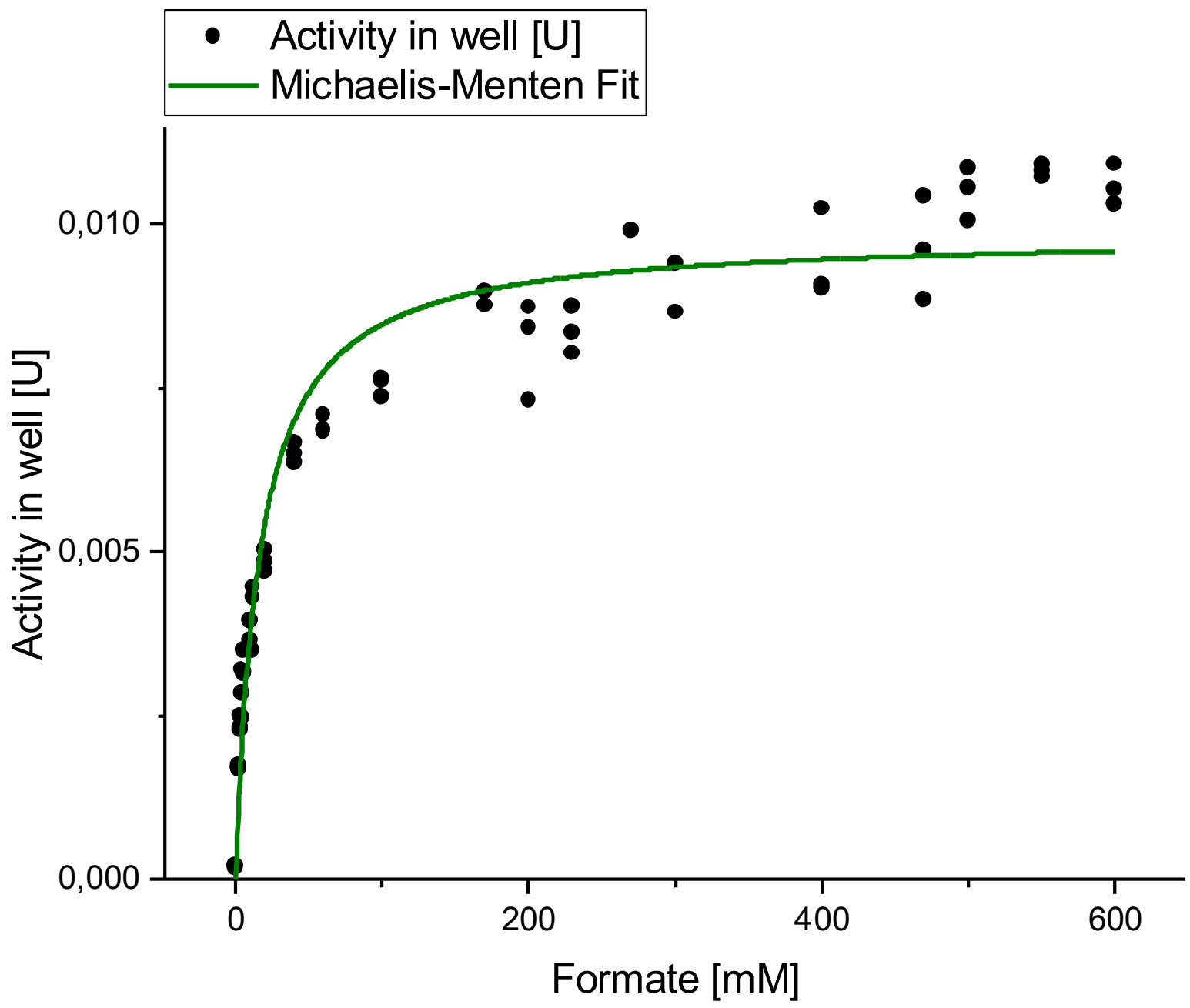

Formate kinetics for CbFDH-C23S were recorded as follows: maximal velocity V_{max} was determined to be 0.0099±1.70E-4 U and K_M was calculated to be 16.2±1.43 mM, with an R^2 value of 0.95 for the plot.

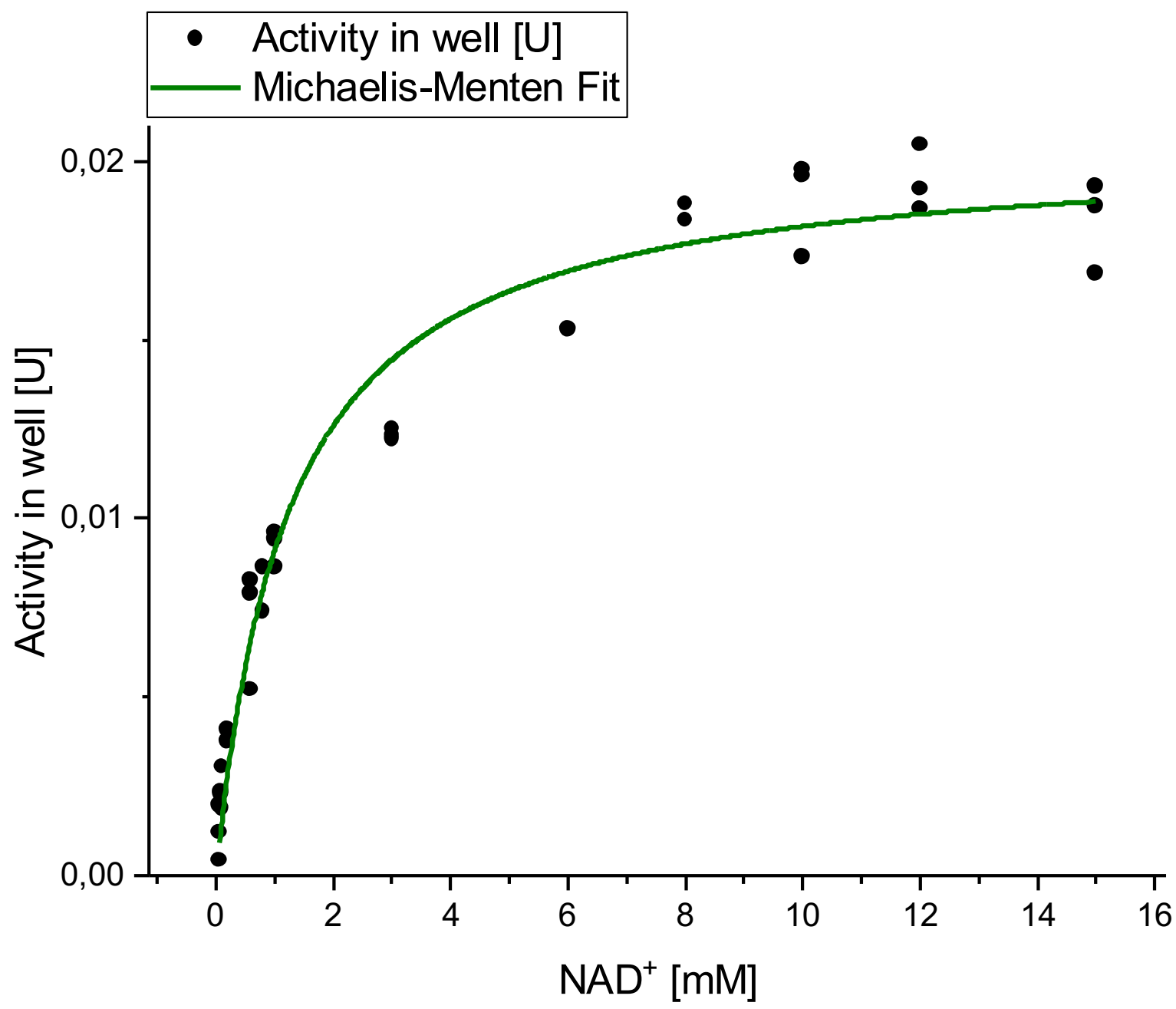

On the other hand, NAD^+ kinetics for CbFDH-C23S were: maximal velocity V_{max} = 0.020±1.70E-4 U and K_M was calculated to be 1.24±0.13 mM, with an R^2 value of 0.97.

S4.4.3 CbFDH-C23S/F285D

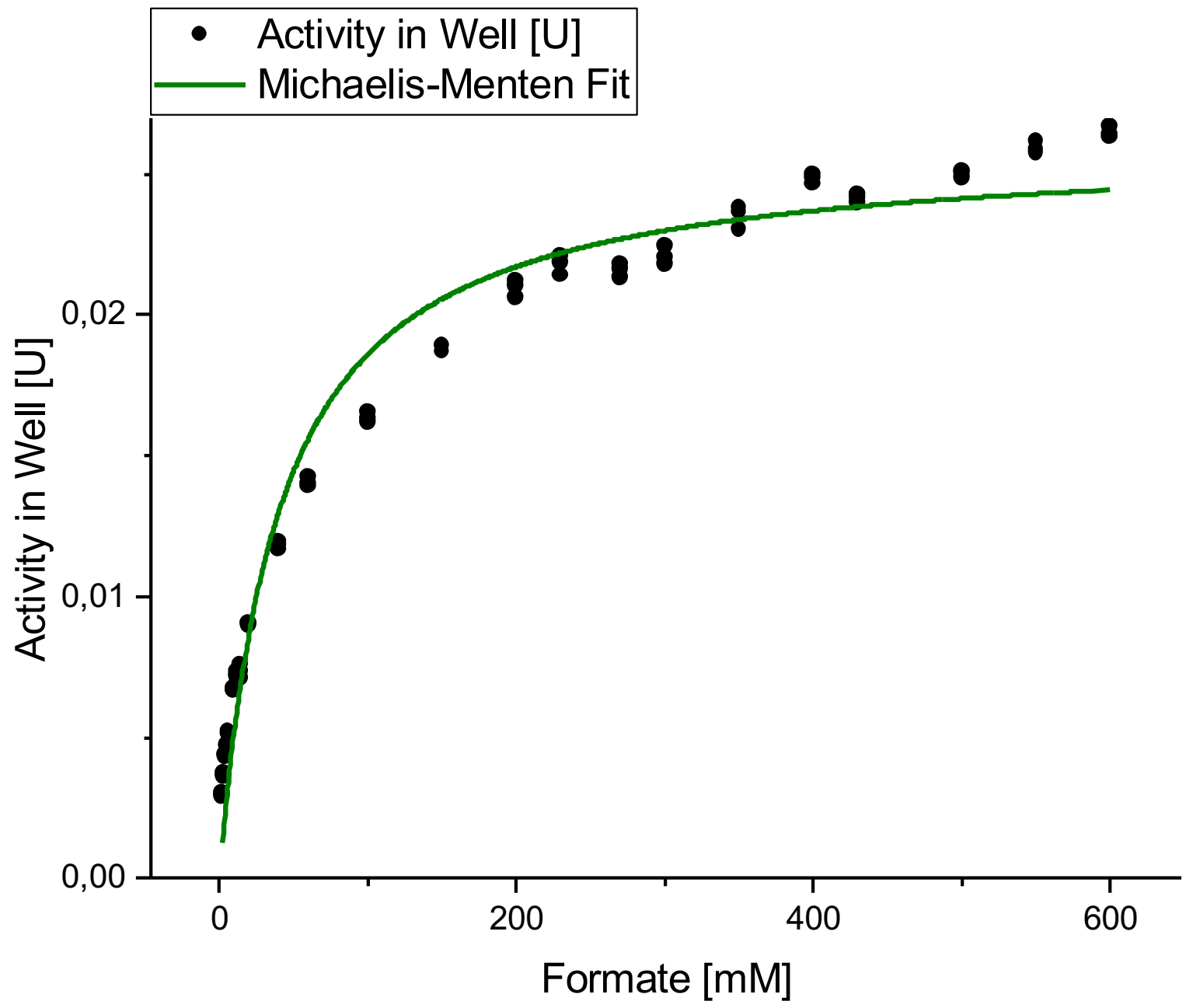

Formate kinetics for CbFDH-C23S/F285D were: maximal velocity V_{max} was determined to be 0.026±3.88E-4 U and K_M was calculated to be 37.6±3.35 mM, with an R^2 value of 0.96.

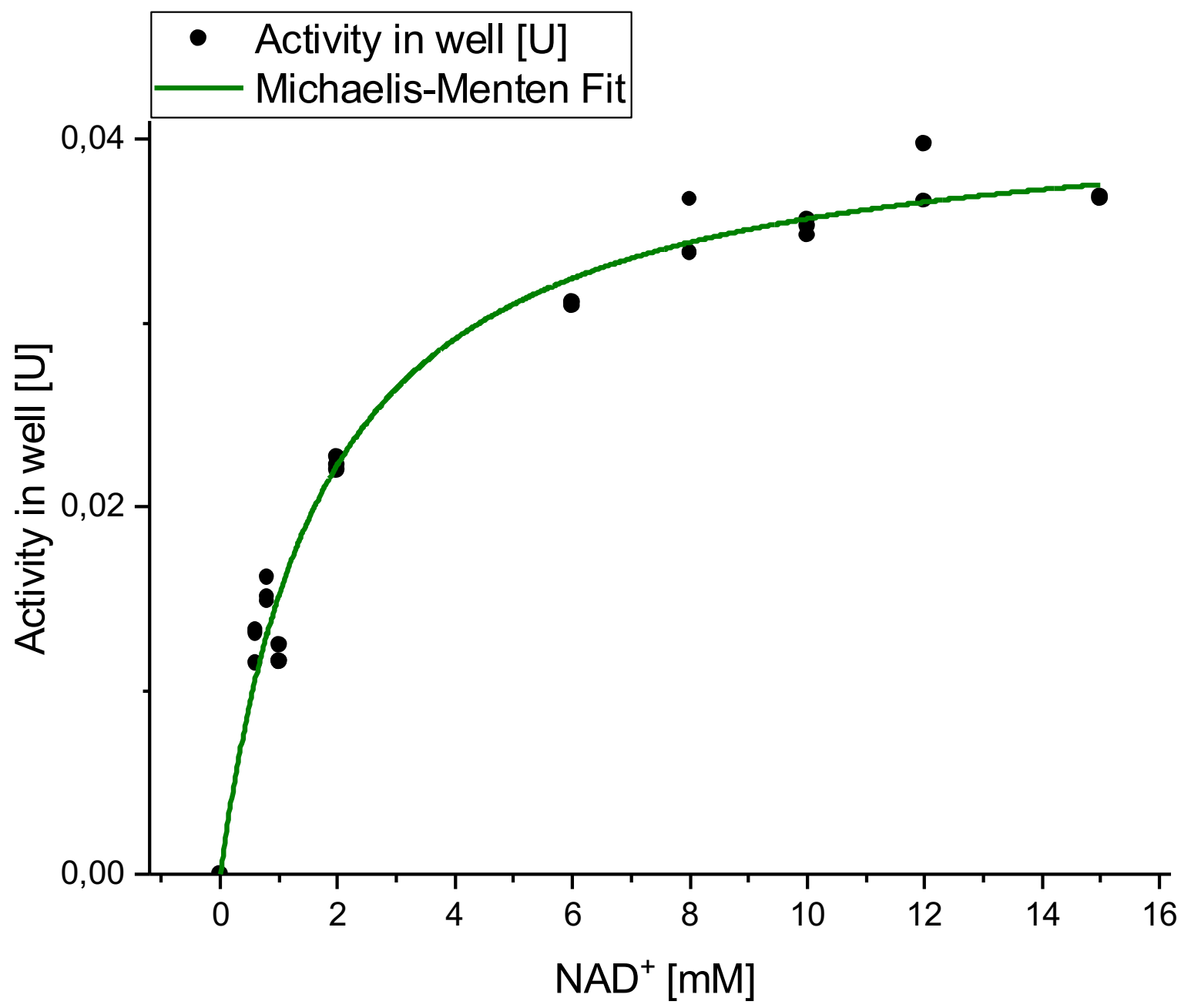

NAD^+ kinetics for CbFDH-C23S/F285D were: maximal velocity V_{max} = 0.0419±9.47E-4 U and K_M was calculated to be 1.76±0.15 mM, with an R^2 value of 0.97.

S4.4.4 CbFDH-C23S/P286K

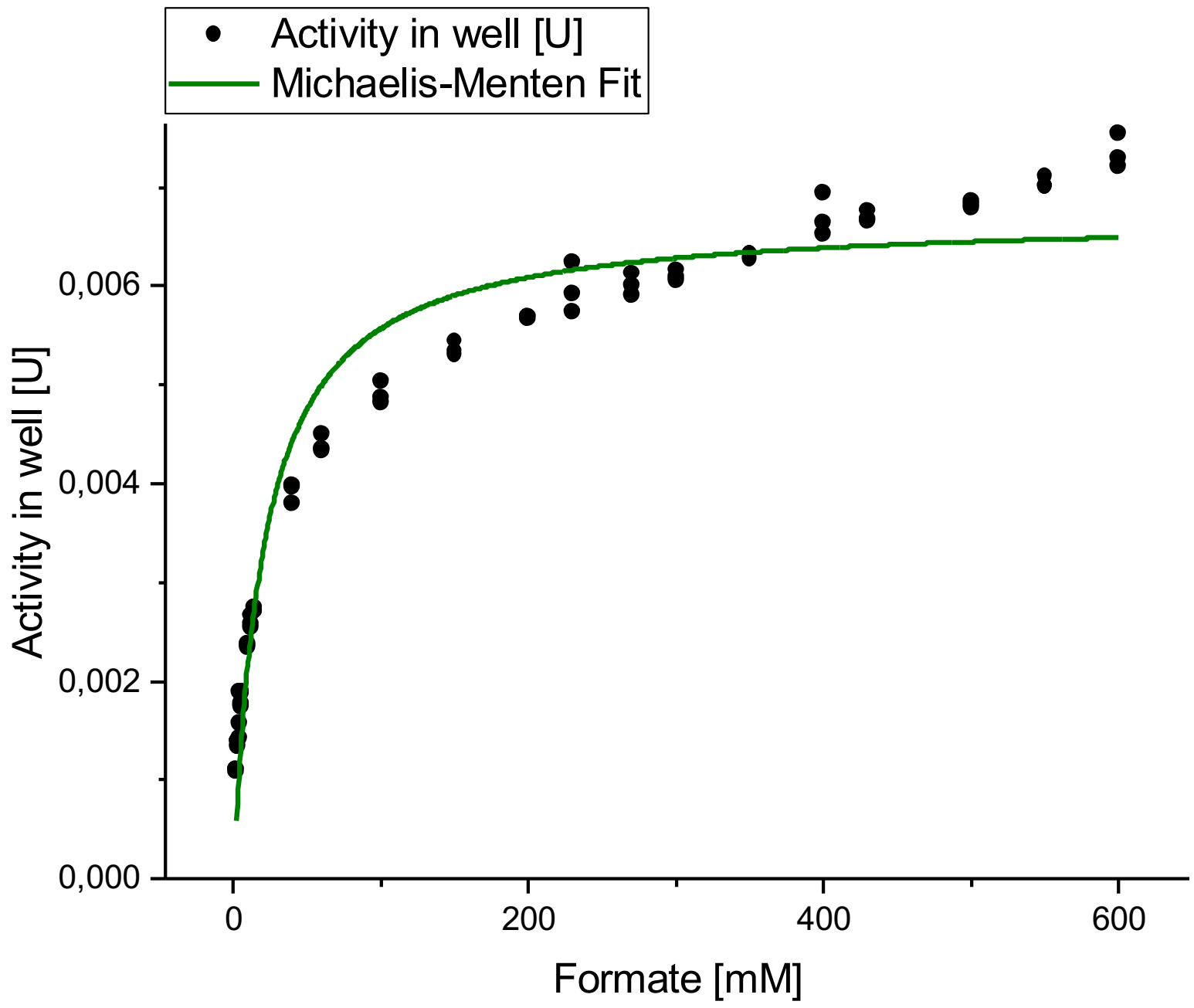

Formate kinetics for CbFDH-C23S/P286K were: maximal velocity V_{max} was determined to be 0.0067±9.77E-5 U and K_M was calculated to be 20.5±1.62 mM, with an R^2 value of 0.95.

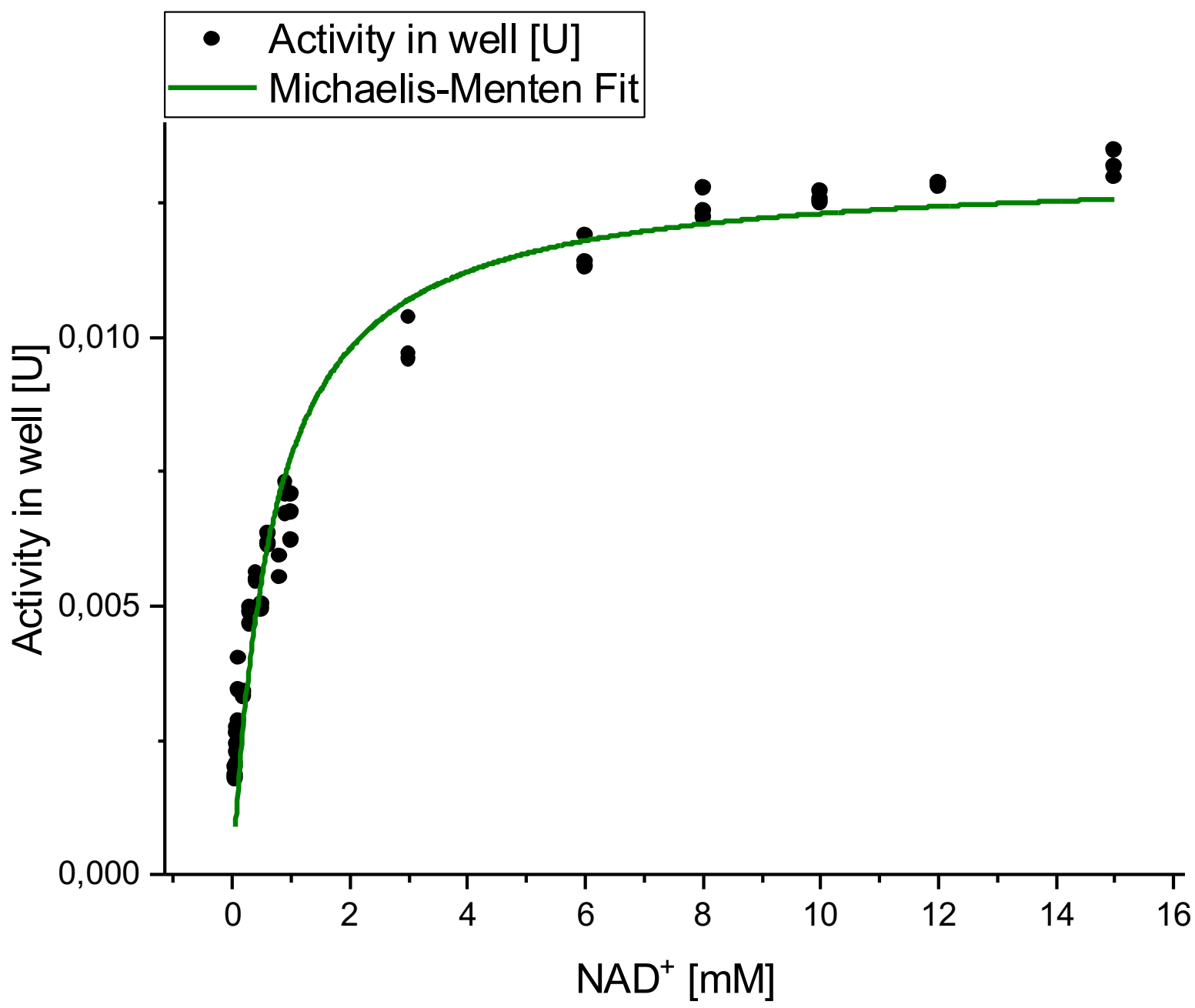

NAD^+ kinetics for CbFDH-C23S/P286K were: maximal velocity V_{max} = 0.0131±2.81E-4 U and K_M was calculated to be 0.68±0.05 mM, with an R^2 value of 0.95.

S4.4.5 CbFDH-C23S/F285D/P286K

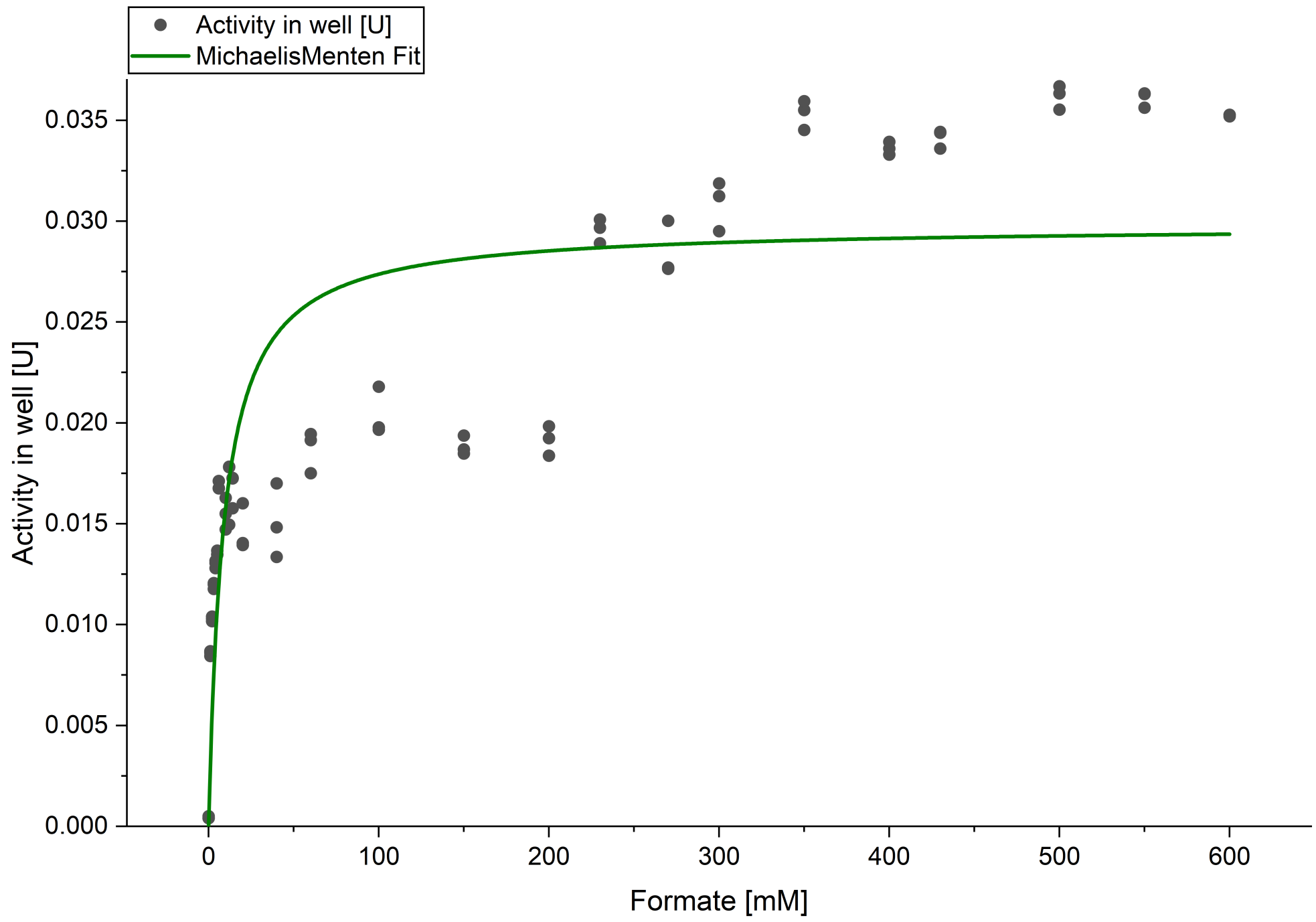

Formate kinetics for CbFDH-C23S/F285D/P286K were: maximal velocity V_{max} was determined to be 0.0298±9.99E-4 U and K_M was calculated to be 8.81±1.60 mM, with an R^2 value of 0.70.

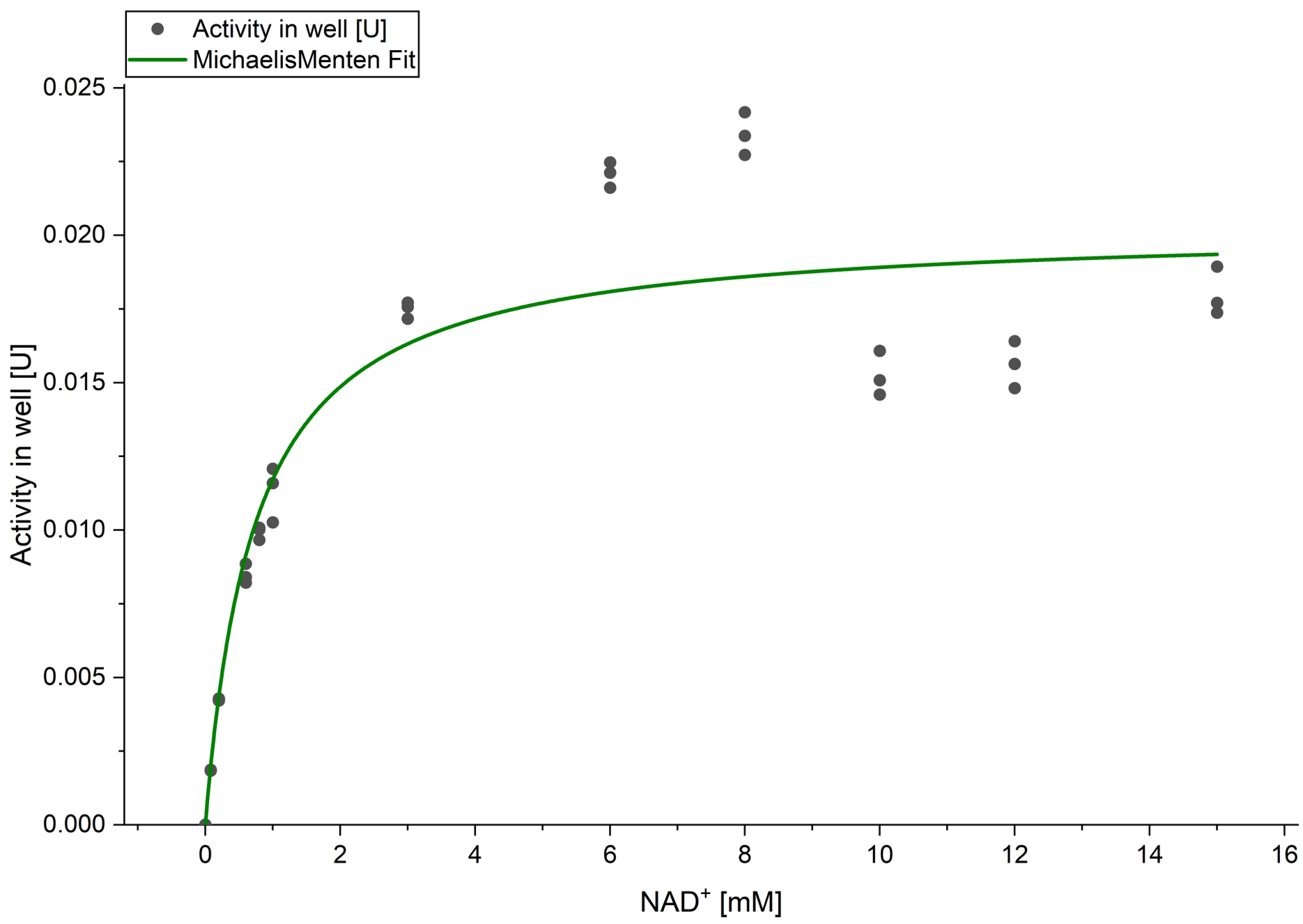

NAD^+ kinetics for CbFDH-C23S/F285D/P286K were: maximal velocity V_{max} = 0.0203±8.34E-4 U and K_M was calculated to be 0.73±0.14 mM, with an R^2 value of 0.89.

S4.5 Computational tools

S4.5.1 AlphaFold3

These are the sequences that were uploaded unto AlphaFold3[3] to get the mutation analysis. The obtained simulated structures were downloaded in .cif formate and they were visualised using PyMol (see S4.5.2). The simulations were also studied online in the AlphaFold3 server, including the interactions between residues and the conformational changes incurred from each mutation.

CbFDH-C23S

MKIVLVLYDAGKHAADEEKLYGSTENKLGIANWLKDQGHELITTSDKEGGNSVLDQHIPDADIIITTPFHPAYITKER
IDKAKKLKLVVVAGVGSDHIDLDYINQTGKKISVLEVTGSNVVSVAEHVLMTMLVLVRNFVPAHEQIINHDWEVAAI
AKDAYDIEGKTIATIGAGRIGYRVLERLVPFNPKELLYYDYQALPKDAEEKVGARRVENIEELVAQADIVTINAPLHA
GTKGLINKELLSKFKKGAWLVNTARGAICVAEDVAAALESGQLRGYGGDVWFPQPAPKDHPWRDMRNKYGAGN
AMTPHYSGTTLDAQTRYAEGTKNILESFFTGKFDYRPQDIILLNGEYITKAYGKHDKK

S4.5.2 CbFDH-C23S/F285D

MKIVLVLYDAGKHAADEEKLYGSTENKLGIANWLKDQGHELITTSDKEGGNSVLDQHIPDADIIITTPFHPAYITKER
IDKAKKLKLVVVAGVGSDHIDLDYINQTGKKISVLEVTGSNVVSVAEHVLMTMLVLVRNFVPAHEQIINHDWEVAAI
AKDAYDIEGKTIATIGAGRIGYRVLERLVPFNPKELLYYDYQALPKDAEEKVGARRVENIEELVAQADIVTINAPLHA
GTKGLINKELLSKFKKGAWLVNTARGAICVAEDVAAALESGQLRGYGGDVWDPQPAPKDHPWRDMRNKYGAG
NAMTPHYSGTTLDAQTRYAEGTKNILESFFTGKFDYRPQDIILLNGEYITKAYGKHDKK

S4.5.3 CbFDH-C23S/P286K

MKIVLVLYDAGKHAADEEKLYGSTENKLGIANWLKDQGHELITTSDKEGGNSVLDQHIPDADIIITTPFHPAYITKER
IDKAKKLKLVVVAGVGSDHIDLDYINQTGKKISVLEVTGSNVVSVAEHVLMTMLVLVRNFVPAHEQIINHDWEVAAI
AKDAYDIEGKTIATIGAGRIGYRVLERLVPFNPKELLYYDYQALPKDAEEKVGARRVENIEELVAQADIVTINAPLHA
GTKGLINKELLSKFKKGAWLVNTARGAICVAEDVAAALESGQLRGYGGDVWFKQPAPKDHPWRDMRNKYGAGN
AMTPHYSGTTLDAQTRYAEGTKNILESFFTGKFDYRPQDIILLNGEYITKAYGKHDKK

S4.5.4 CbFDH-C23S/F285D/P286K

MKIVLVLYDAGKHAADEEKLYGSTENKLGIANWLKDQGHELITTSDKEGGNSVLDQHIPDADIIITTPFHPAYITKER
IDKAKKLKLVVVAGVGSDHIDLDYINQTGKKISVLEVTGSNVVSVAEHVLMTMLVLVRNFVPAHEQIINHDWEVAAI
AKDAYDIEGKTIATIGAGRIGYRVLERLVPFNPKELLYYDYQALPKDAEEKVGARRVENIEELVAQADIVTINAPLHA
GTKGLINKELLSKFKKGAWLVNTARGAICVAEDVAAALESGQLRGYGGDVWDKQPAPKDHPWRDMRNKYGAG
NAMTPHYSGTTLDAQTRYAEGTKNILESFFTGKFDYRPQDIILLNGEYITKAYGKHDKK

S4.5.1 PyMol

PyMol[4] was used to visualise the crystal structures for CbFDH (apo, PDB: 5DNA; holo, PDB: 5DN9)[1]. These structures were used as the base to study the variant structures compared on this article C23S, C23S/F285D, C23S/F285D and C23S/F285D/P286K by employing the Wizard > Mutagenesis > Protein tool.

Additionally, PyMol was used to visualise the simulated structures of our variants obtained with AlphaFold3 (see S4.5.1), as well as measure the distances between glutamate 48 and proline/lysine 286, as required. This was done through Wizard > Measurements.

S4.6 Application reactions

The application reactions set up using ERED 110 and KRED 459 (from ERED kit and KRED kit, Codexis, Inc.) were set up in the following amounts and the following order, and incubated at 30 °C overnight (18 h), with no shaking. Each reaction was set in duplicate, one containing wild type CbFDH, and the other CbFDH-C23S/F285D. The reactions were tested multiple times, using different conditions, and these are the ones that offered optimum results. The amounts of ERED110 and KRED459 were weighed and taken as close to 5 mg as possible, with a possible error of ±1 mg.

Table S11: Application reactions set up, chemicals added in the order listed

Component	Volume
100 mM KP_i pH 7.0	1 mL
2-Cyclohexenone	5 µL
1 M NaCOOH	51 µL
FDH (3.5 mg/mL)	50 µL
ERED 110	~5 mg
100 mM NAD^+	26 µL

Component	Volume
100 mM KP_i pH 7.0	1 mL
Acetophenone	5 µL
1 M NaCOOH	51 µL
FDH (3.5 mg/mL)	50 µL
KRED 459	~5 mg
100 mM NAD^+	26 µL

The reactions were stopped at the same time, by denaturing the enzymes at 95 °C for 5 minutes. The reactions were centrifuged (13000 rpm, 5 minutes) and the supernatants were transferred into clean glass vials. The aqueous supernatants were extracted with ethyl acetate (3 x 1 mL). The combined organic phases were dried over sodium sulfate anhydrous, filtered over a silica plug, and injected in the GCMS for analysis. Conversion rates were calculated from the GCMS data (see below).

S4.6.1 ERED 110 + Wild Type FDH

Conversion percentage (obtained through peak area integration): 91%

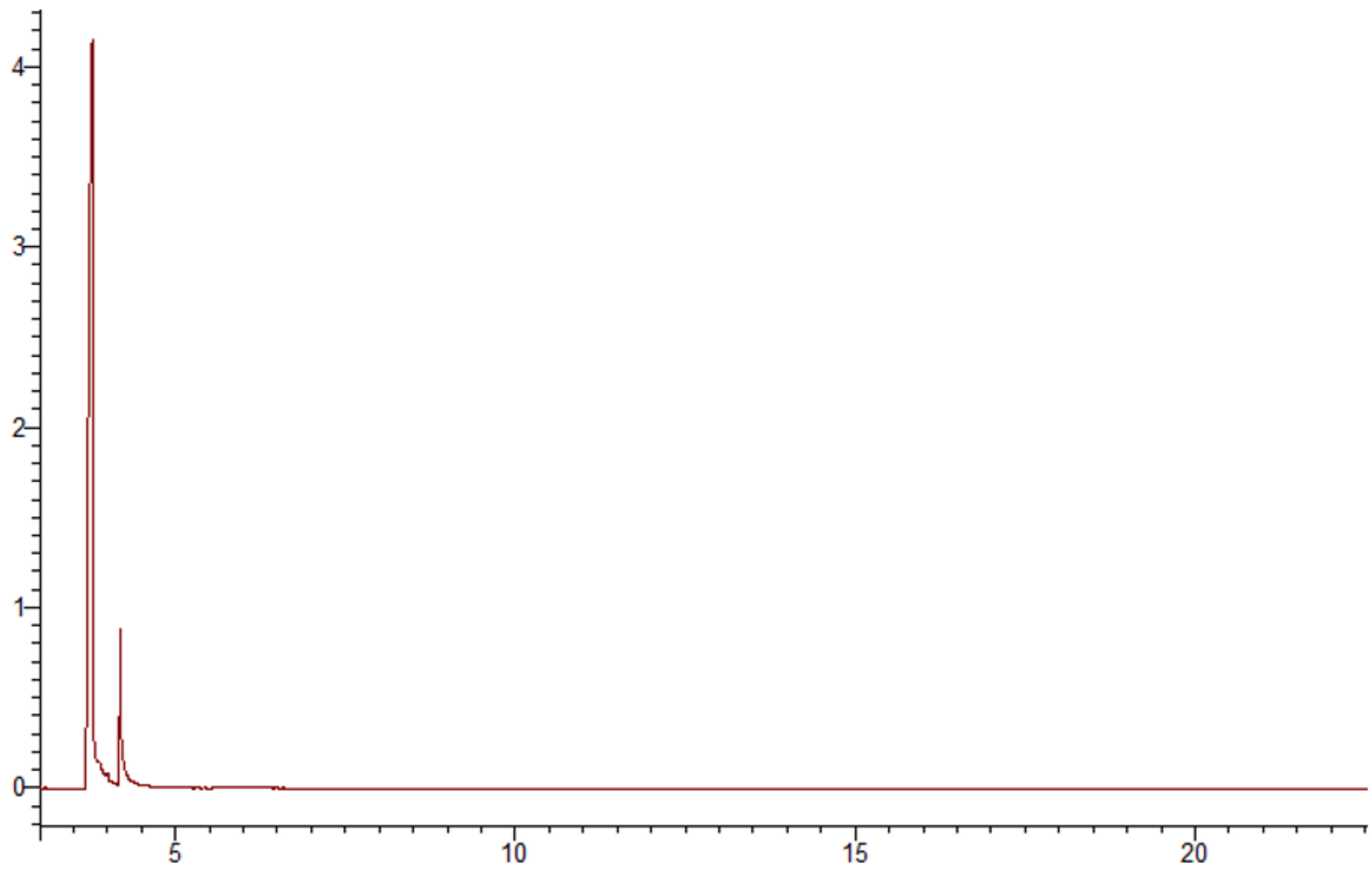

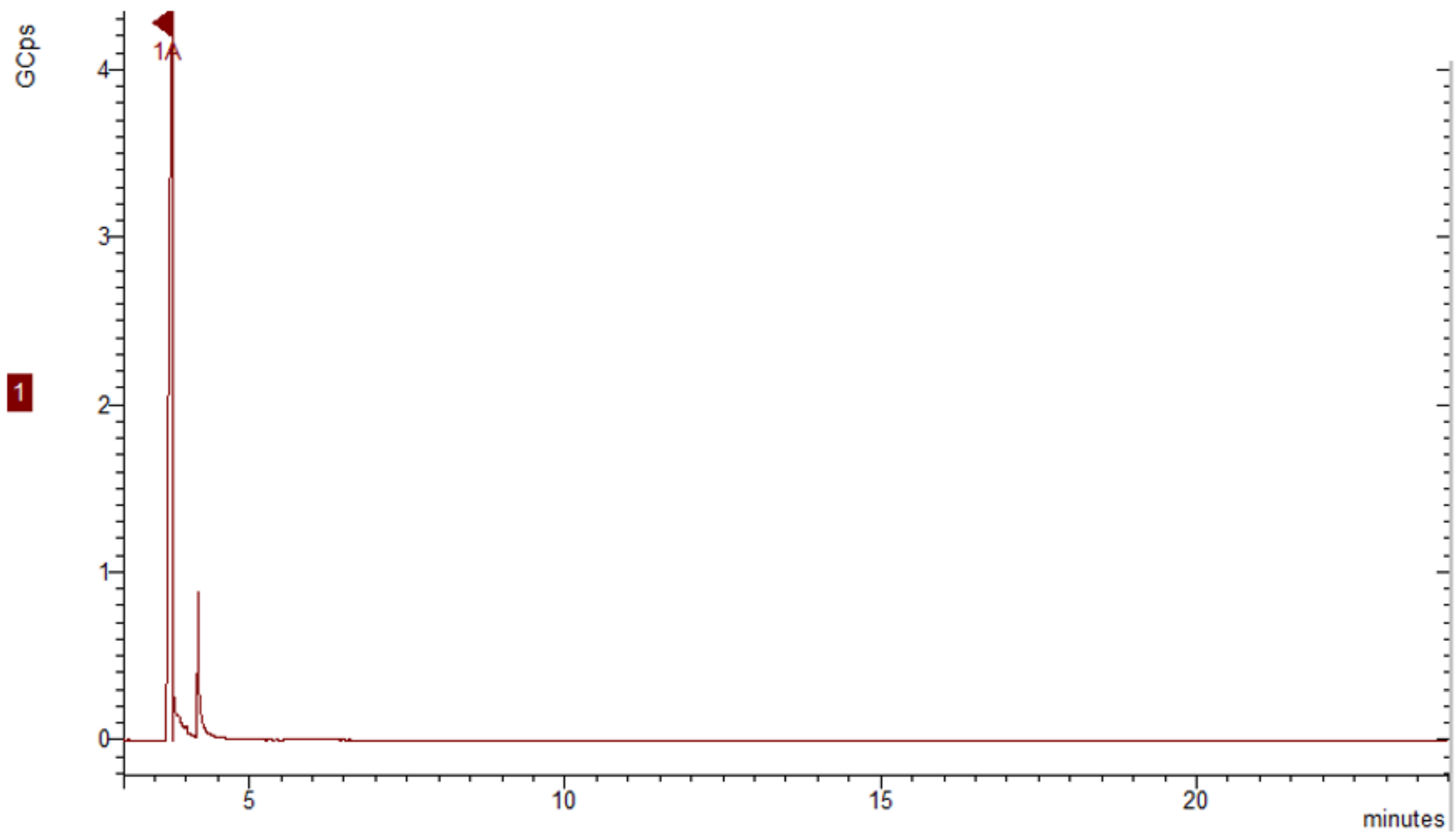

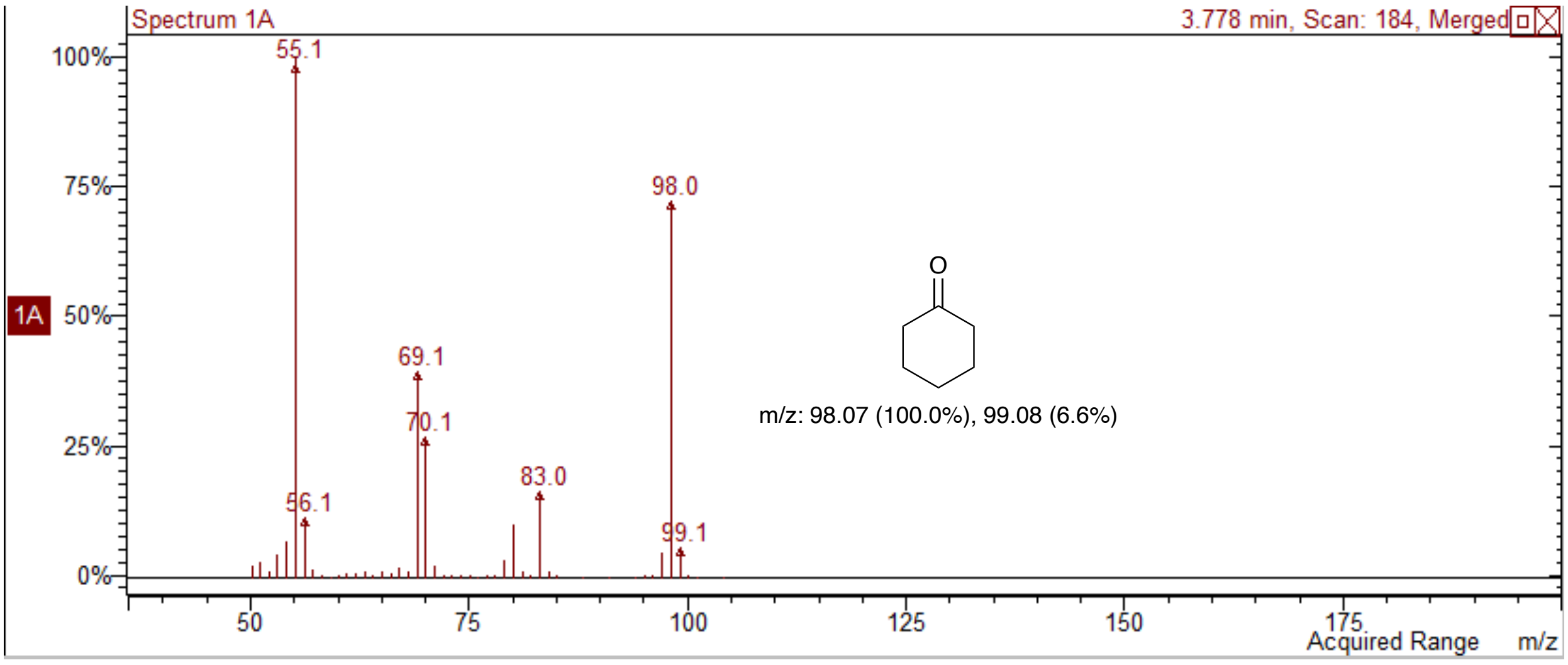
Spectrum 1A
3.778 min, Scan: 184, Merged
55.1
98.0
69.1
70.1
83.0
56.1
99.1
m/z: 98.07 (100.0%), 99.08 (6.6%)
Acquired Range
m/z

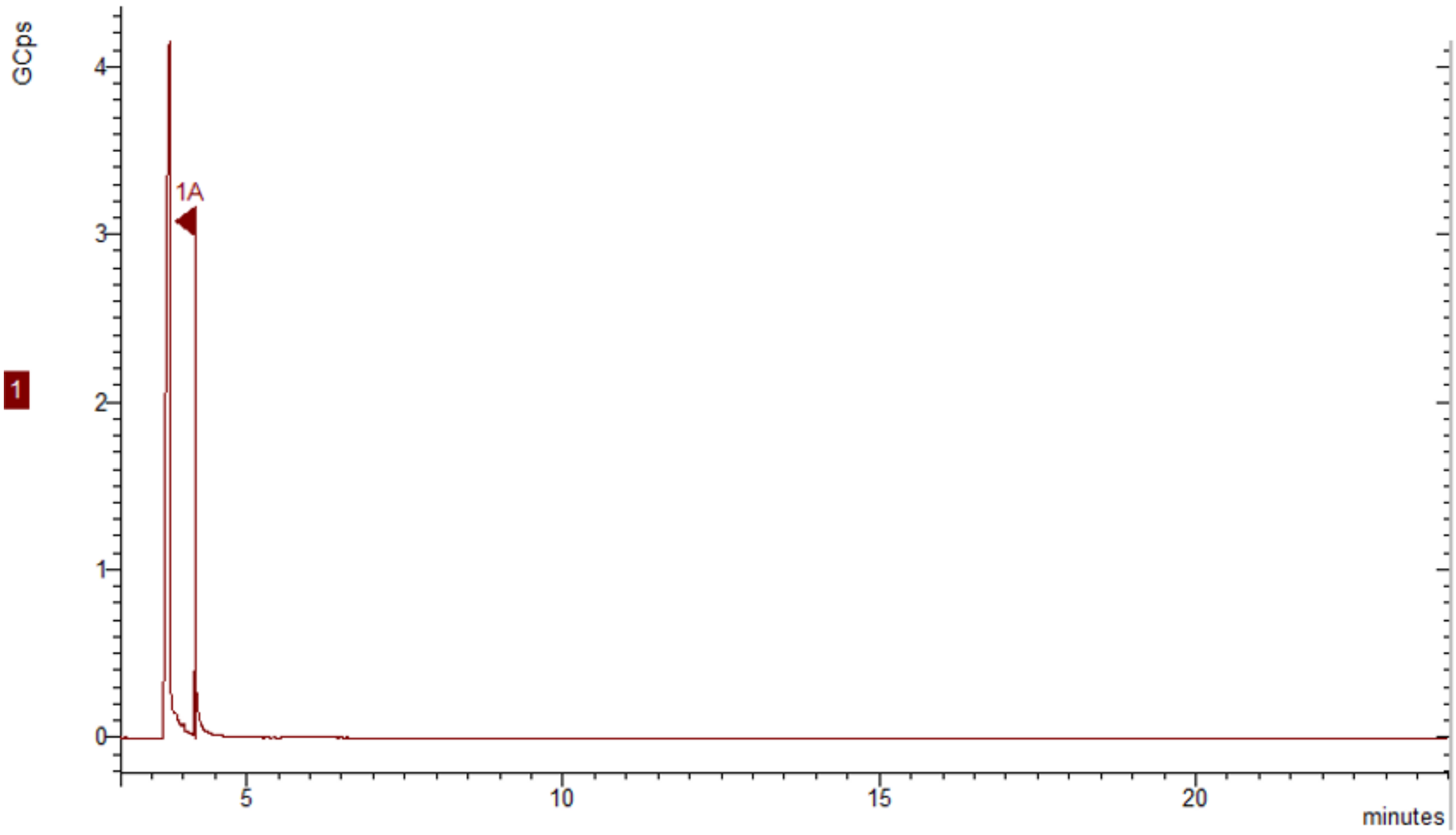
GCps
1A
minutes

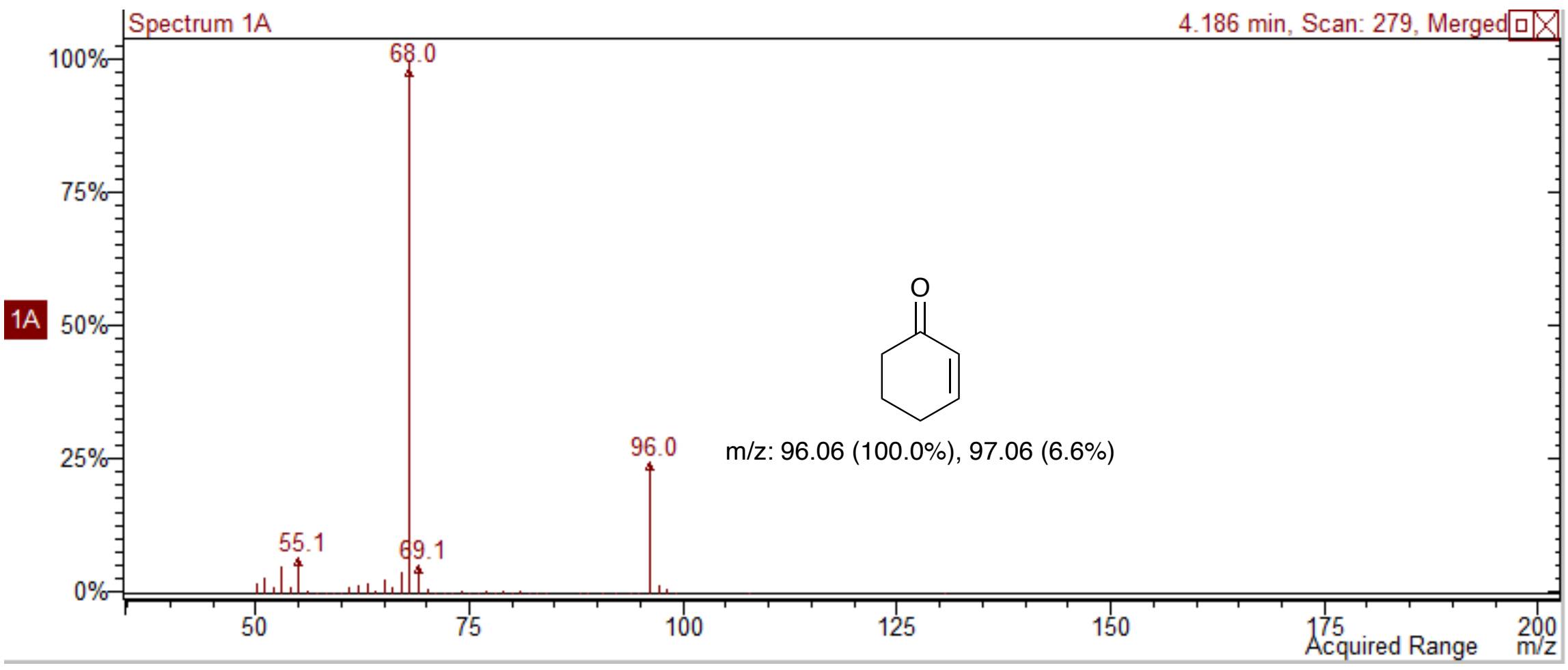
Spectrum 1A
4.186 min, Scan: 279, Merged
68.0
96.0
55.1
69.1
m/z: 96.06 (100.0%), 97.06 (6.6%)
Acquired Range
m/z

S4.6.2 ERED 110 + CbFDH-C23S/F285D

Conversion percentage (obtained through peak area integration): 98%

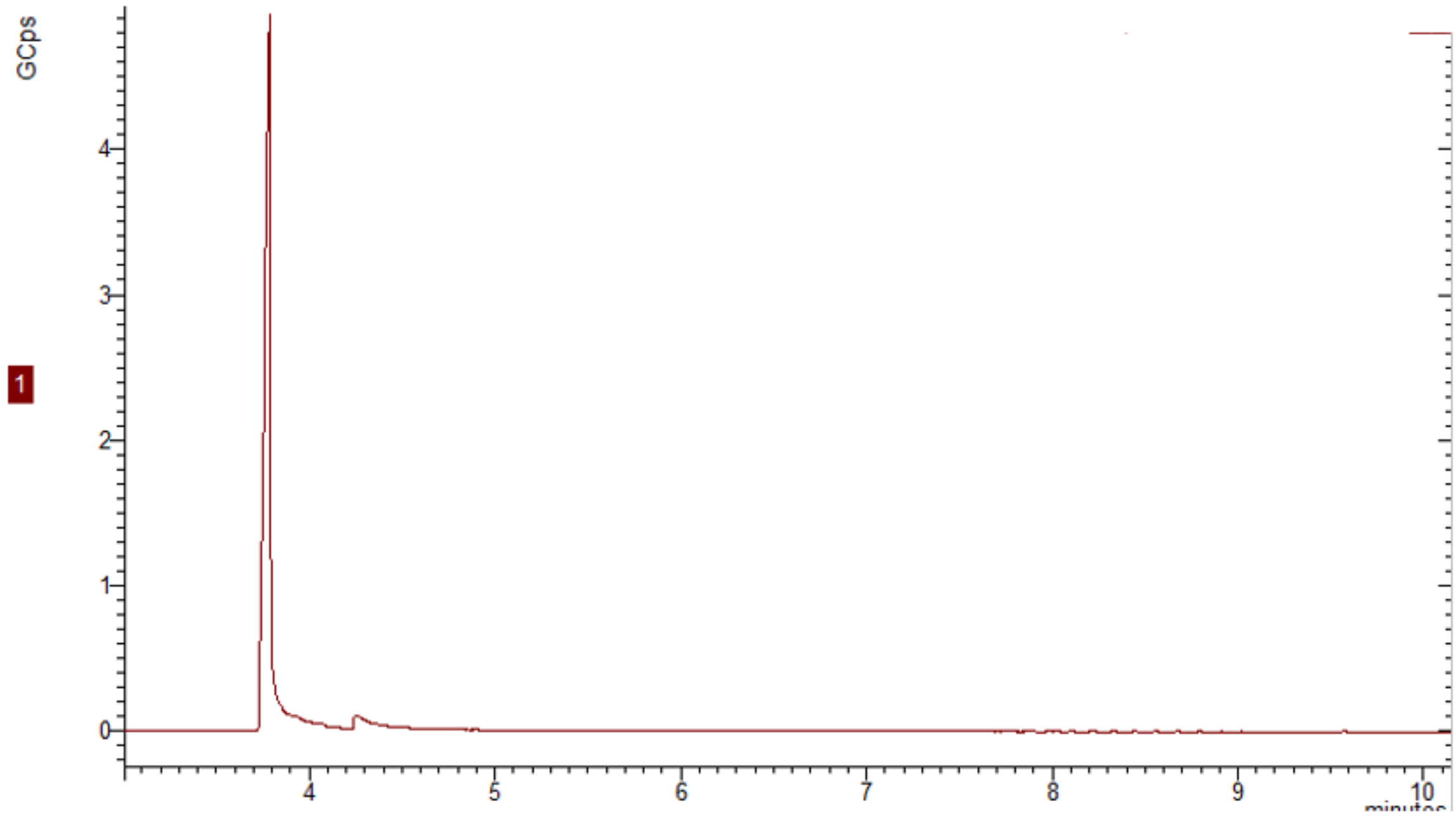

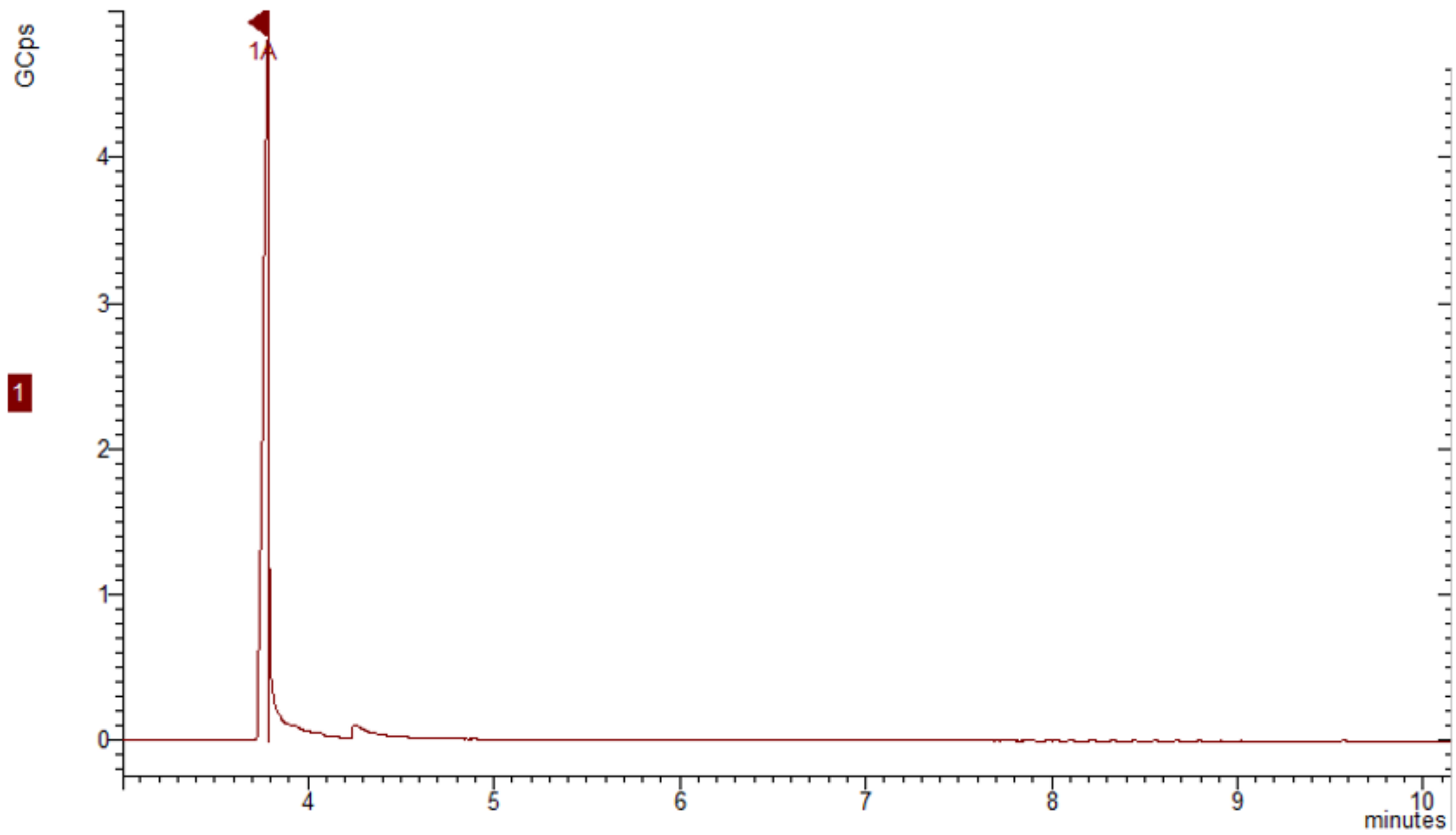

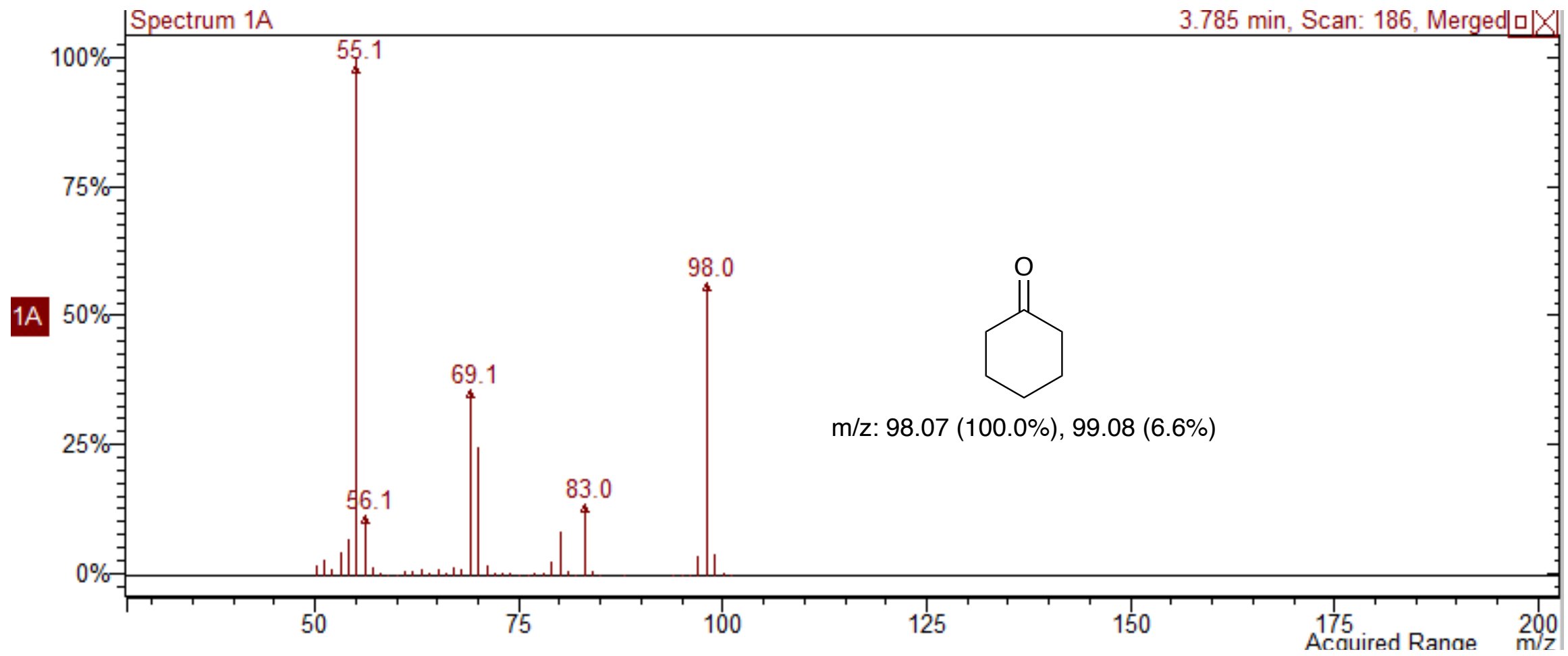
Spectrum 1A
3.785 min, Scan: 186, Merged
100%
75%
1A 50%
25%
0%
55.1
56.1
69.1
83.0
98.0
O
m/z: 98.07 (100.0%), 99.08 (6.6%)
50
75
100
125
150
175
200
Acquired Range
m/z

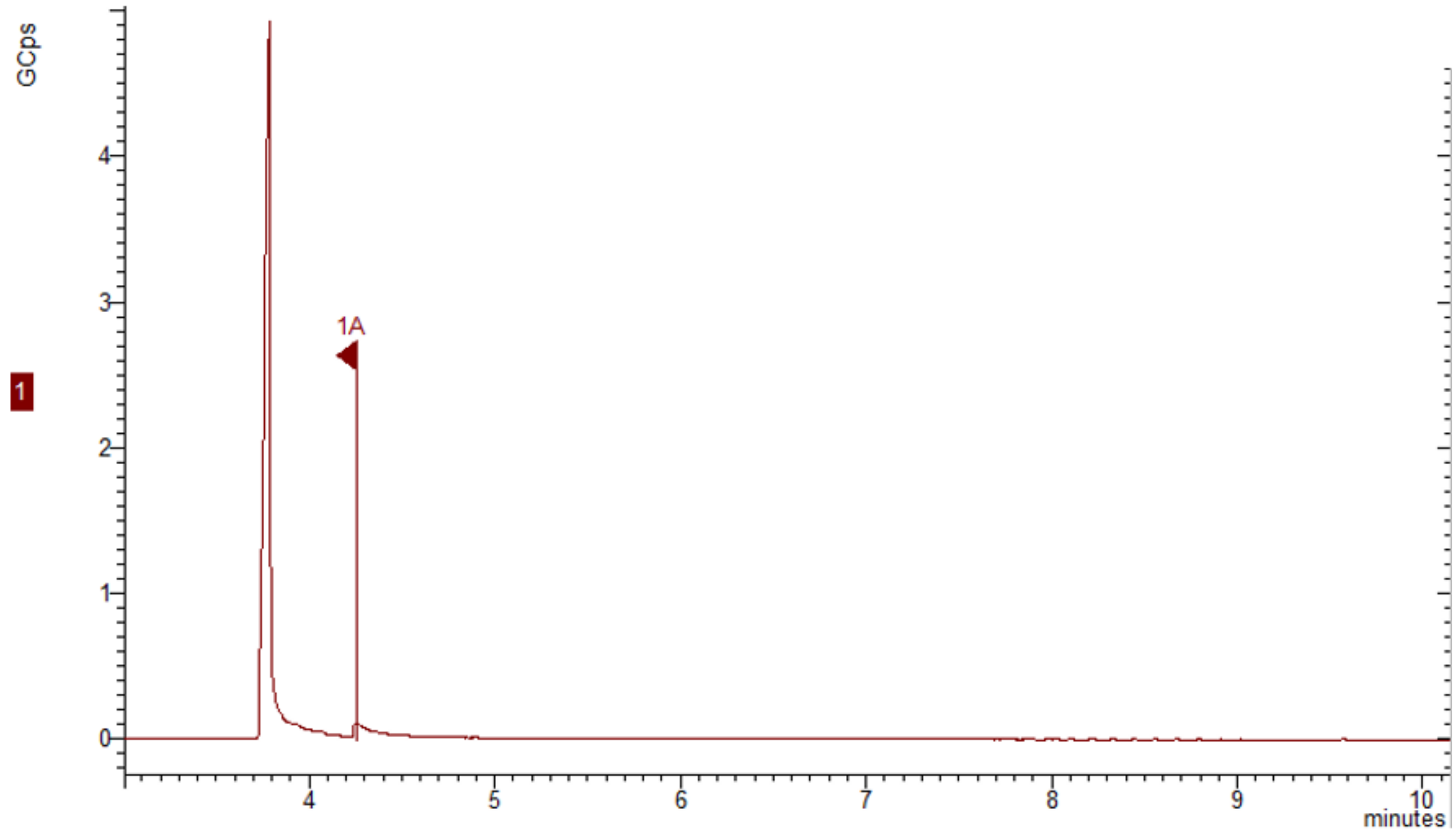
GCps
4
3
2
1
0
1
1A
4
5
6
7
8
9
10
minutes

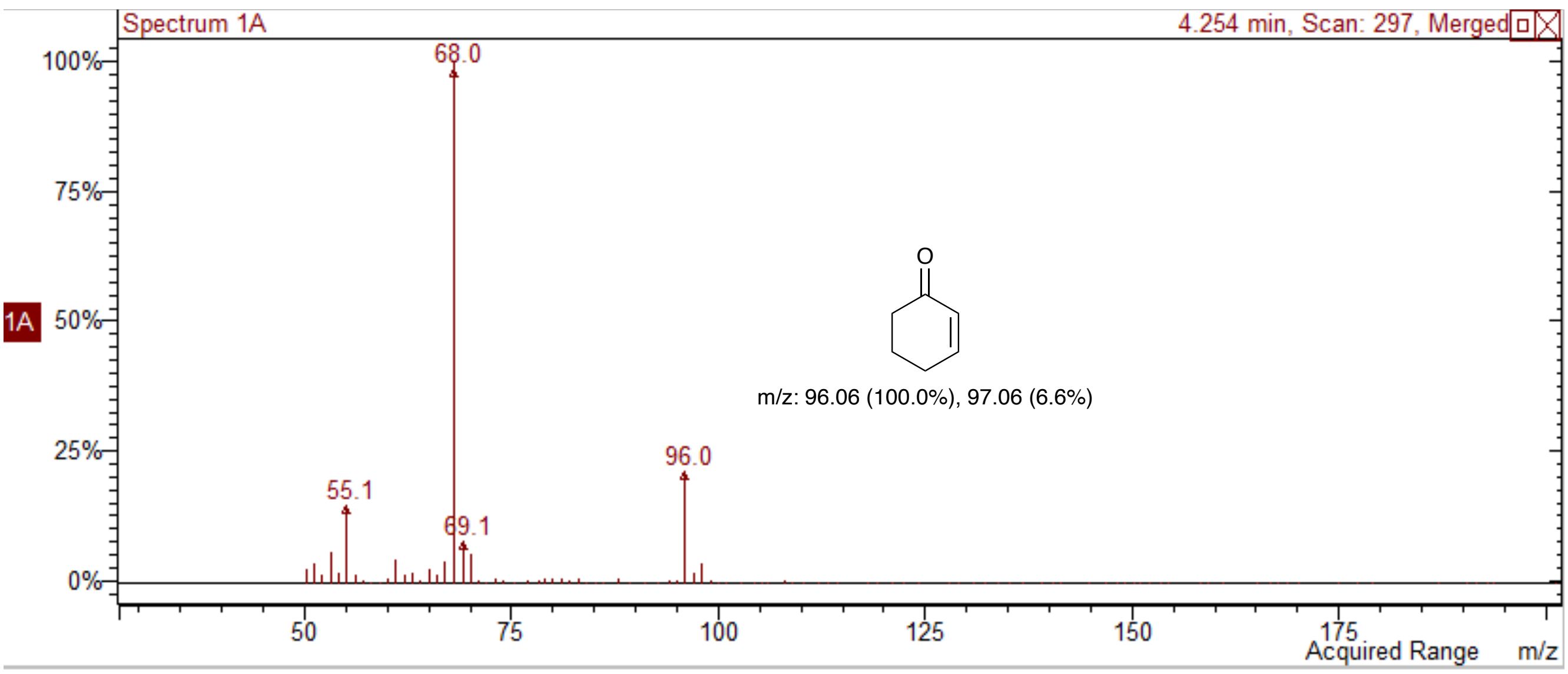
Spectrum 1A
4.254 min, Scan: 297, Merged
100%
75%
1A 50%
25%
0%
68.0
55.1
69.1
96.0
O
m/z: 96.06 (100.0%), 97.06 (6.6%)
50
75
100
125
150
175
Acquired Range
m/z

S4.6.3 KRED 459 + Wild Type FDH

Conversion percentage (obtained through peak area integration): 37%

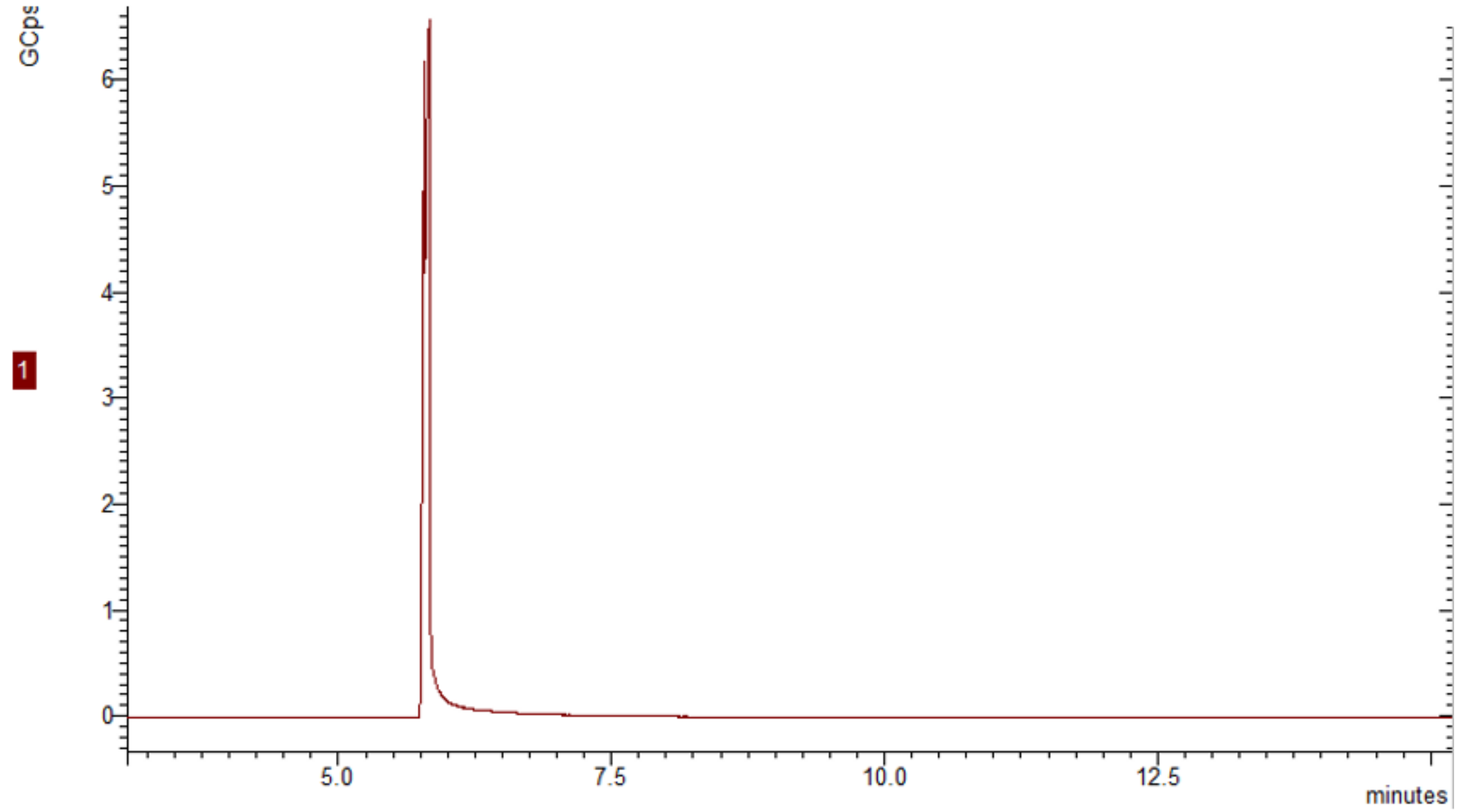

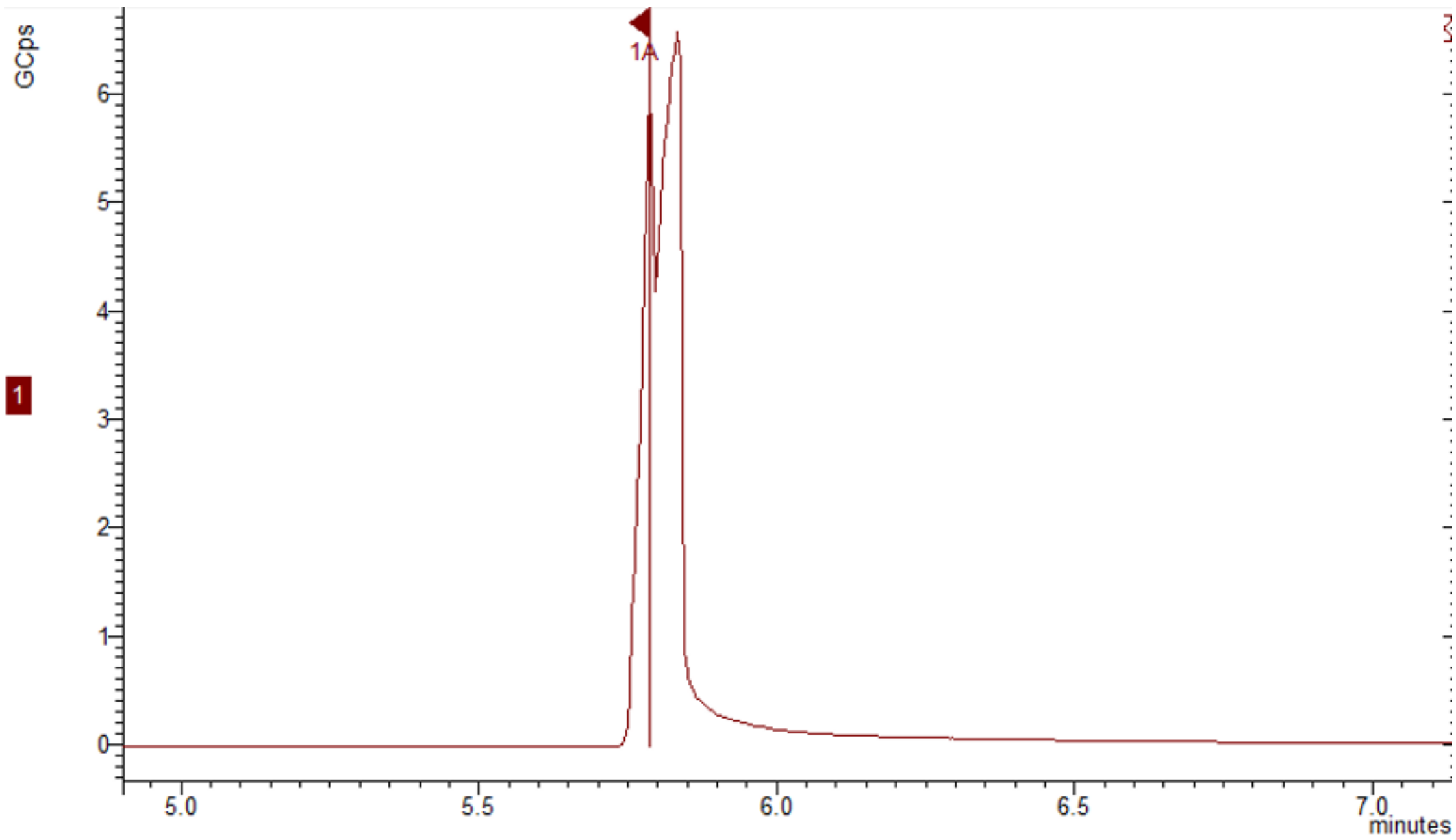

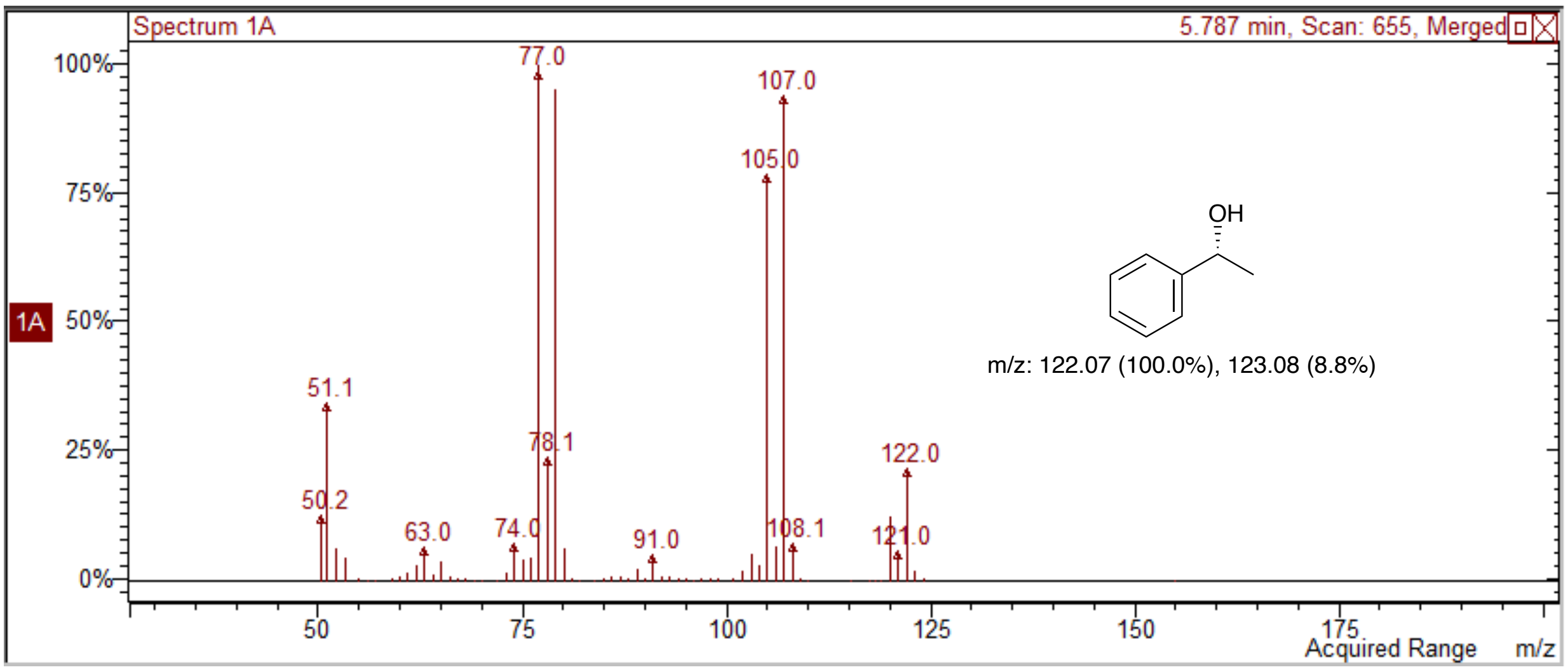
Spectrum 1A
5.787 min, Scan: 655, Merged
100%
75%
1A
50%
25%
0%
77.0
107.0
105.0
51.1
78.1
122.0
50.2
63.0
74.0
91.0
108.1
121.0
OH
m/z: 122.07 (100.0%), 123.08 (8.8%)
50
75
100
125
150
175
Acquired Range
m/z

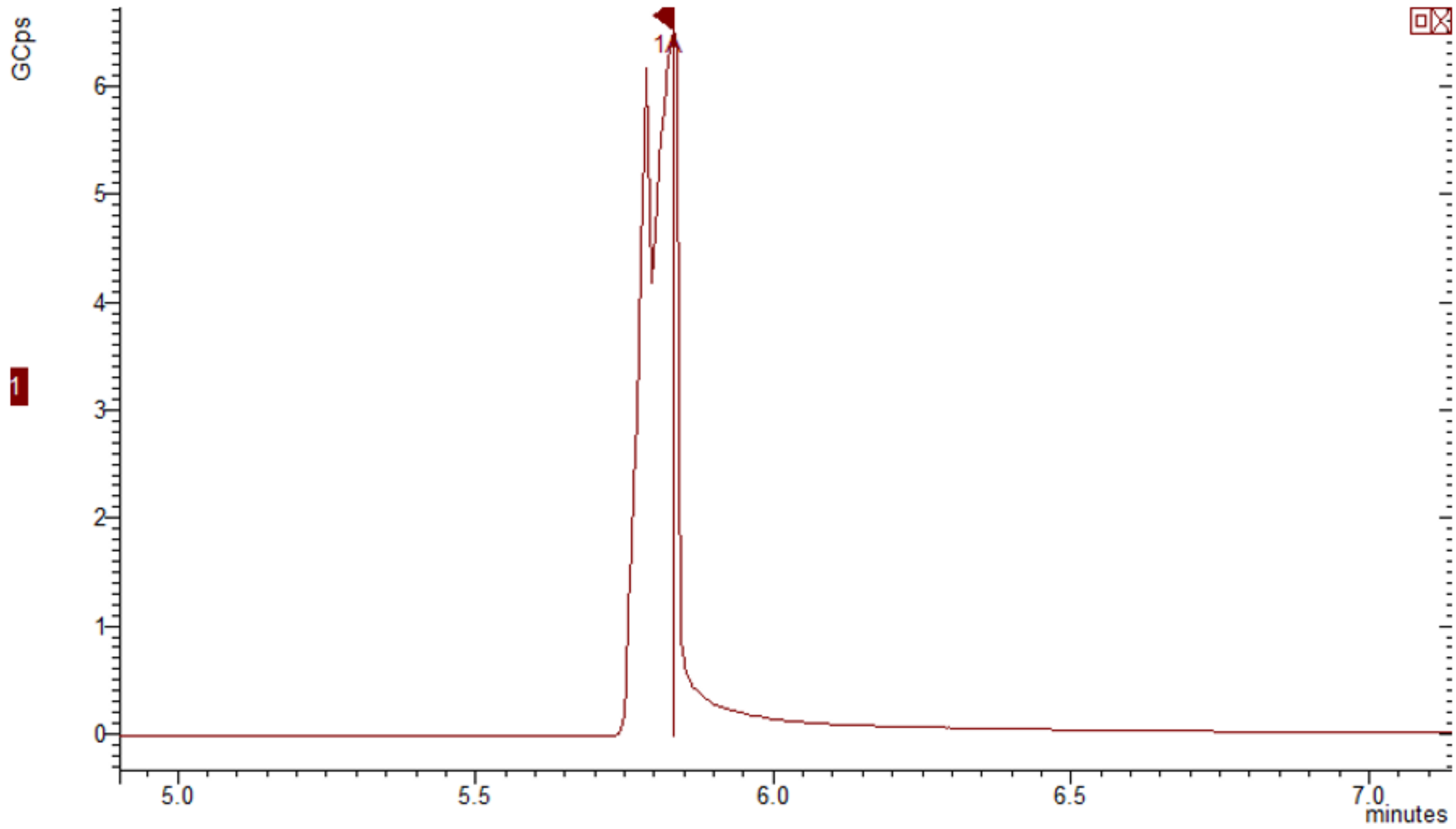
GCps
6
5
4
3
2
1
0
1
5.0
5.5
6.0
6.5
7.0
minutes

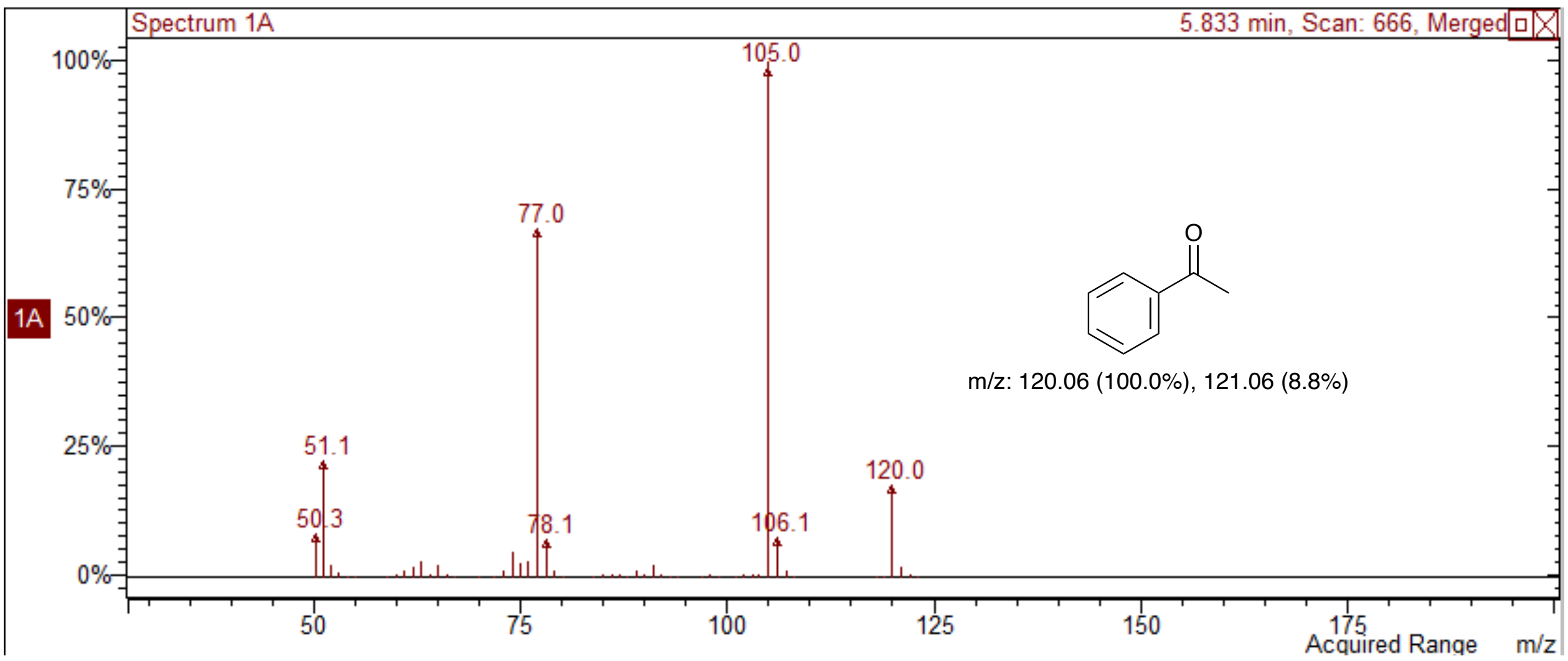
Spectrum 1A
5.833 min, Scan: 666, Merged
100%
75%
1A
50%
25%
0%
105.0
77.0
51.1
120.0
50.3
78.1
106.1
O
m/z: 120.06 (100.0%), 121.06 (8.8%)
50
75
100
125
150
175
Acquired Range
m/z

S4.6.4 KRED 459 + CbFDH-C23S/F285D

Conversion percentage (obtained through peak area integration): 97%

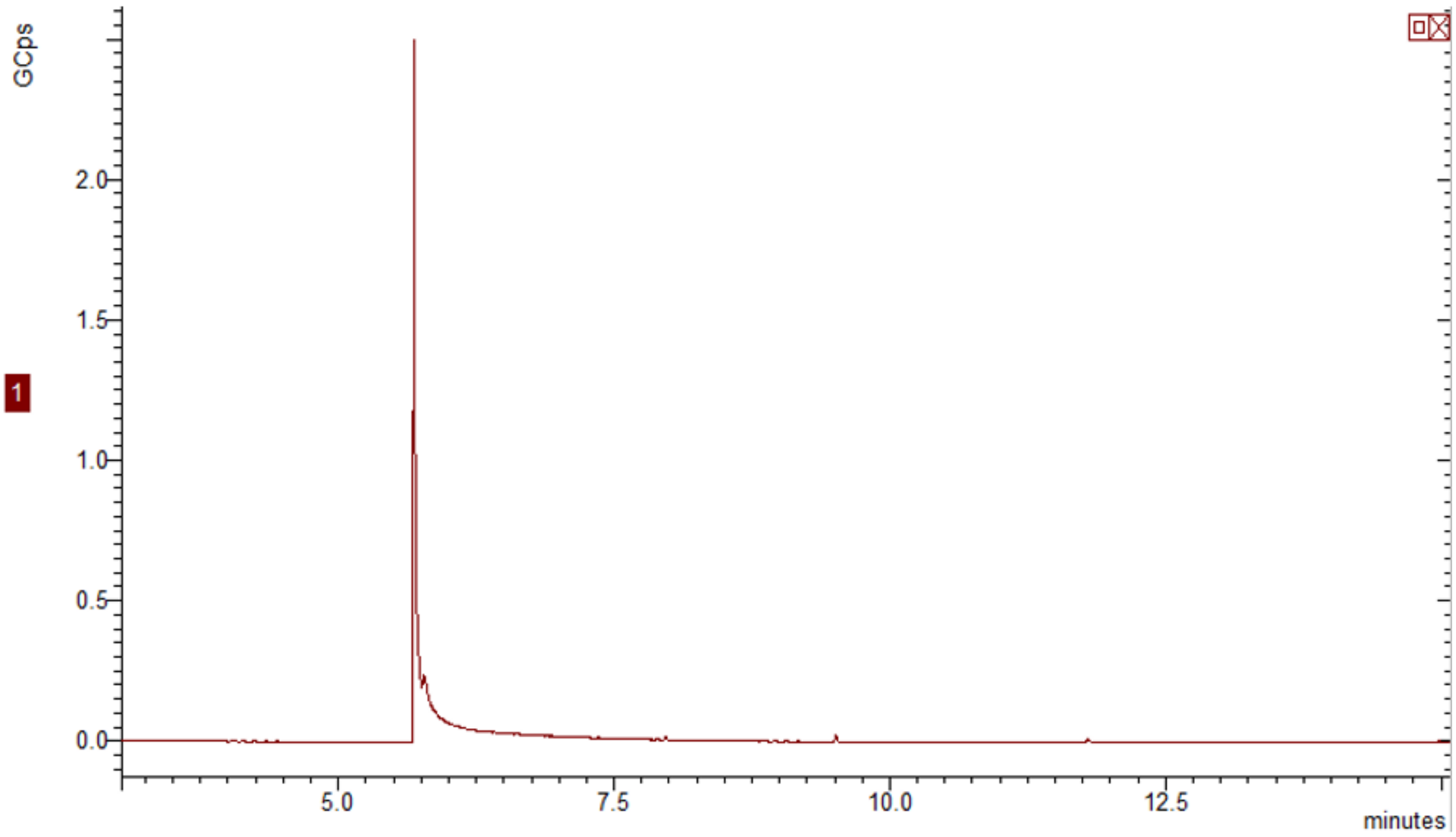

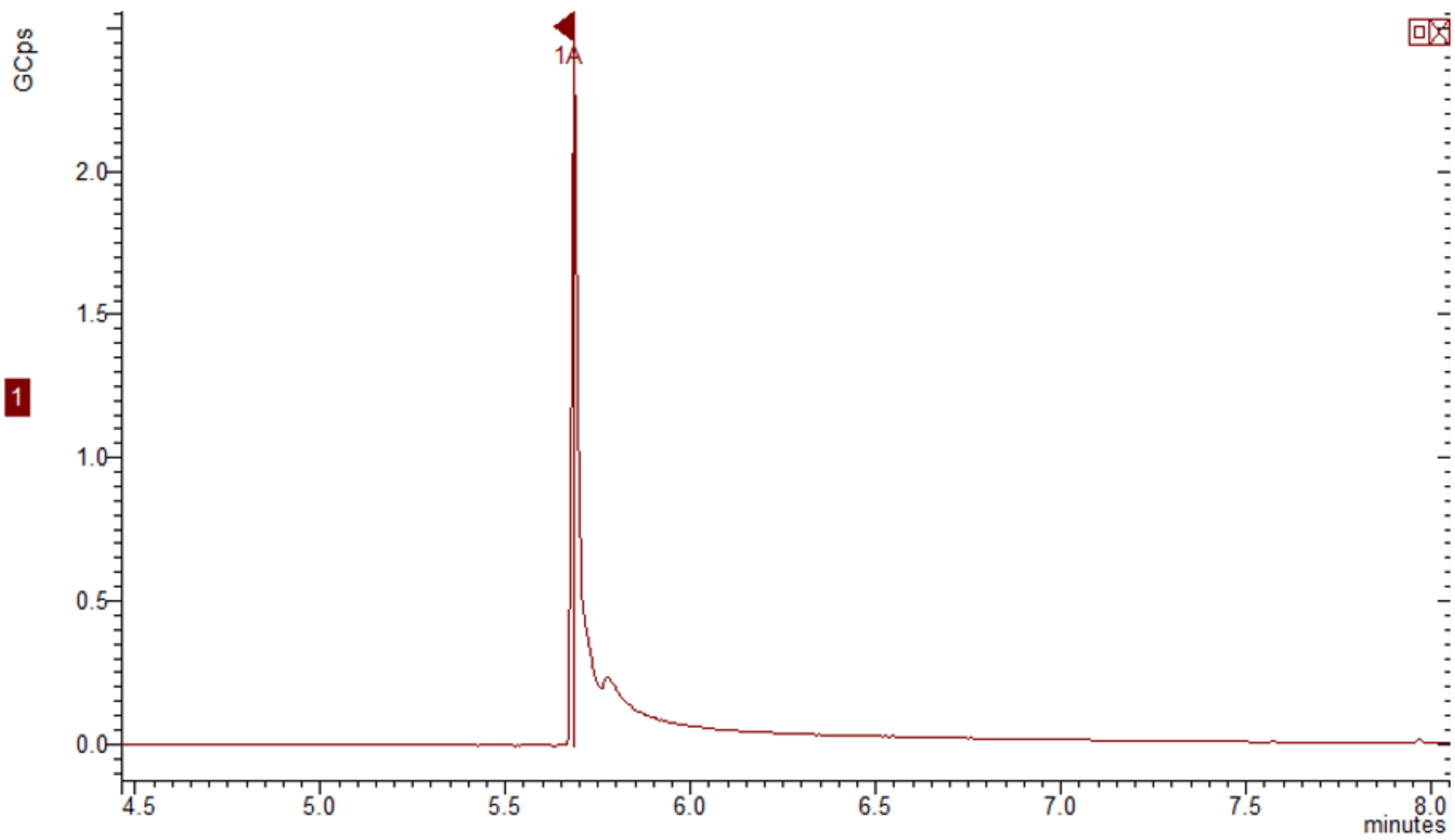

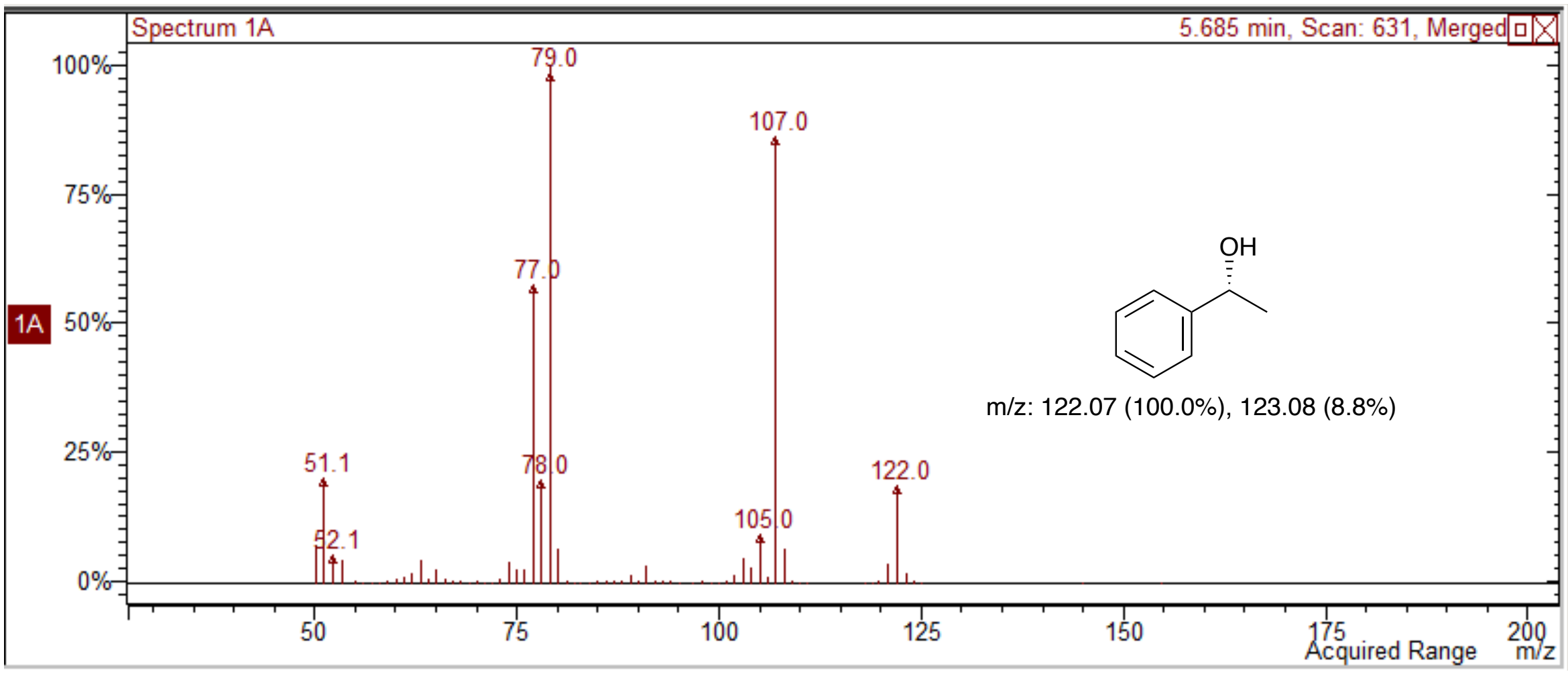
Spectrum 1A
5.685 min, Scan: 631, Merged
100%
75%
50%
25%
0%
1A
79.0
107.0
77.0
78.0
51.1
52.1
105.0
122.0
OH
m/z: 122.07 (100.0%), 123.08 (8.8%)
50
75
100
125
150
175
200
Acquired Range
m/z

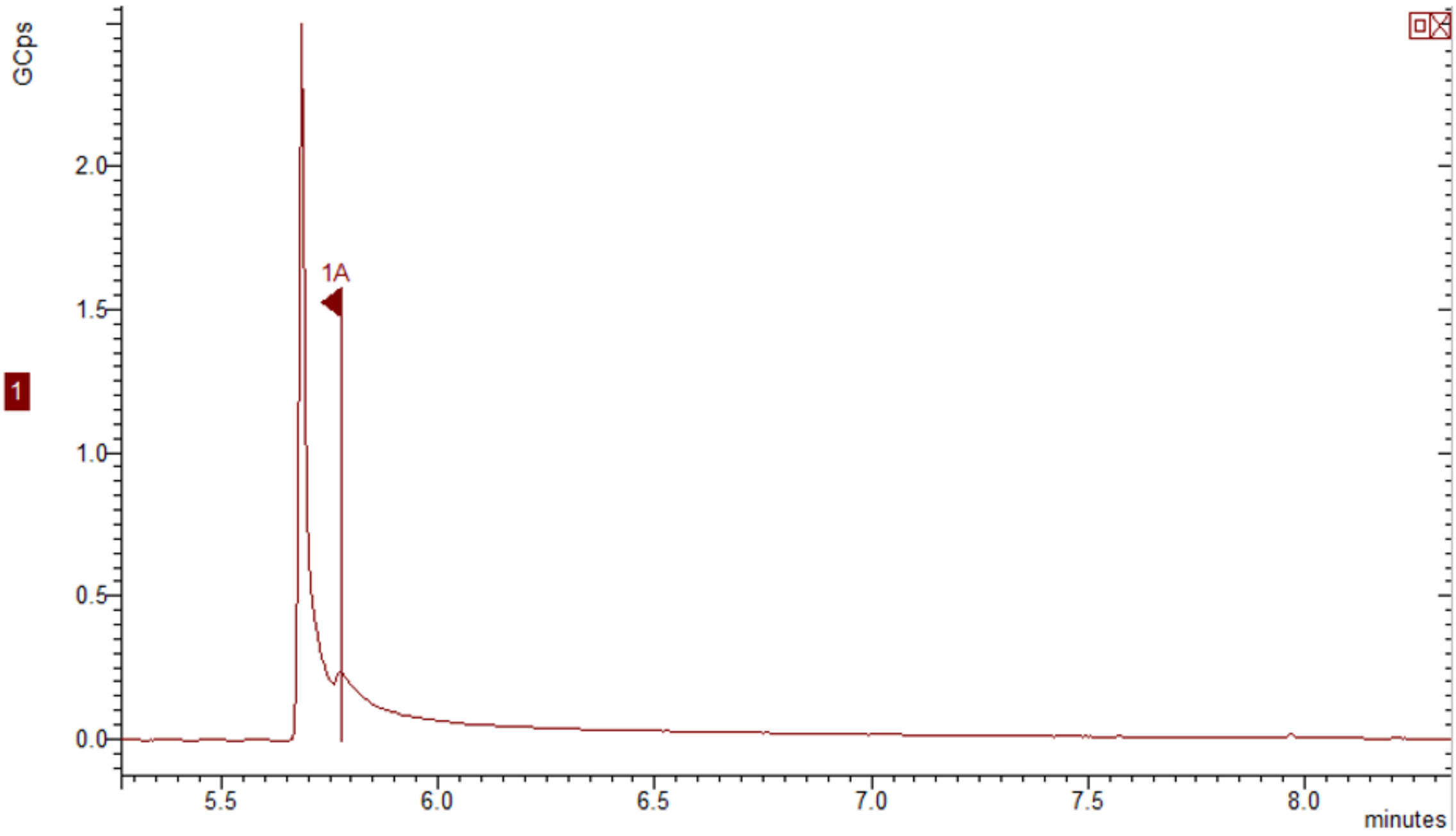
GCps
2.0
1.5
1.0
0.5
0.0
1
1A
5.5
6.0
6.5
7.0
7.5
8.0
minutes

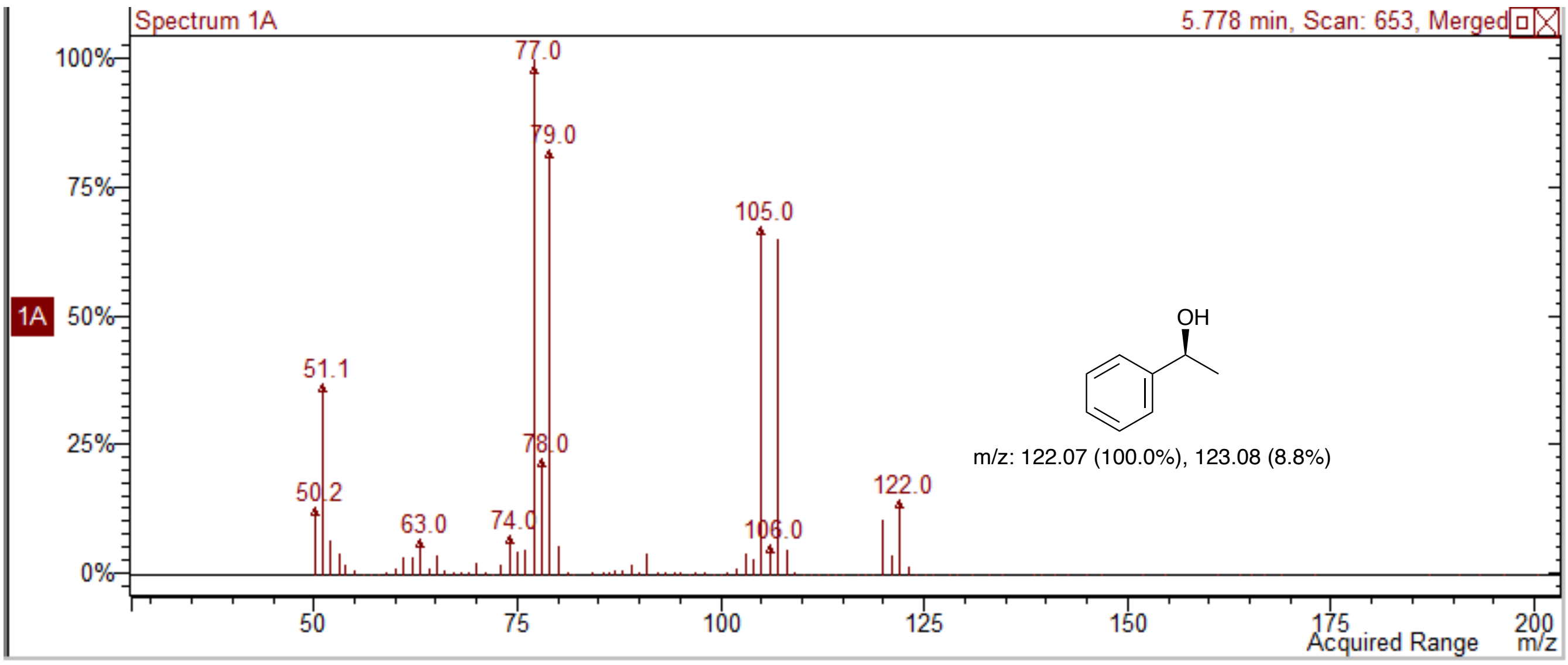
Spectrum 1A
5.778 min, Scan: 653, Merged
100%
75%
50%
25%
0%
1A
77.0
79.0
105.0
51.1
78.0
50.2
63.0
74.0
106.0
122.0
OH
m/z: 122.07 (100.0%), 123.08 (8.8%)
50
75
100
125
150
175
200
Acquired Range
m/z

S5. References

[1] Q. Guo, L. Gakhar, K. Wickersham, K. Francis, A. Vardi-Kilshtain, D. T. Major, C. M. Cheatum, A. Kohen, "Structural and kinetic studies of formate dehydrogenase from *Candida boidinii*" *Biochemistry*, **2016**, *55*(19), 2760.

[2] O. Edelheit, A. Hanukoglu, I. Hanukoglu, "Single and efficient site-directed mutagenesis using two single-primer reactions in parallel to generate mutants for protein structure-function studies" *BMC Biotechnology*, **2009**, *9*, 61.

[3] J. Abramson, J. Adler, J. Dunger, R. Evans, T. Green, A. Pritzel, O. Ronneberger, L. Willmore, A. J. Ballard, J. Bambrick, S. W. Bodenstein, D. A. Evans, C.-C. Hung, M. O'Neill, D. Reiman, K. Tunyasuvunakool, Z. Wu, A. Žemgulitė, E. Arvaniti, C. Beattie, O. Bertolli, A. Bridgland, A. Cherepanov, M. Congreve, A. I. Cowen-Rivers, A. Cowie, M. Figurnov, F. B. Fuchs, H. Gladman, R. Jain, Y. A. Khan, C. M. R. Low, K. Perlin, A. Potapenko, P. Savy, S. Singh, A. Stecula, A. Thillaisundaram, C. Tong, S. Yakneen, E. D. Zhong, M. Zielinski, A. Žídek, V. Bapst, P. Kohli, M. Jaderberg, D. Hassabis, J. M. Jumper, "Accurate structure prediction of biomolecular interactions with AlphaFold3" *Nature*, **2024**, *630*, 493-500.

[4] L. W. DeLano, "PyMol: An open-source molecular graphics tool" *Ccp4 Newsletter on Prot. Crystal.* **2002**, *40*, 82-94.
